# Mechanistic evaluation of Jiu Wei Qing Zhi Gao in non-alcoholic fatty liver disease: insights from network Pharmacology and experimental validation

**DOI:** 10.1186/s41065-025-00427-2

**Published:** 2025-04-12

**Authors:** Qinlei Chen, Qianfeng Hu, Fan Zhang, Weiting Lu, Zheng Yuan, Fei Qiao

**Affiliations:** 1 Department of Infectious Diseases, Jiangsu Province Hospital of Chinese Medicine, Affiliated Hospital of Nanjing University of Chinese Medicine, Nanjing, 210001 China; 2 Nanjing University of Chinese Medicine, Nanjing, China 210046

**Keywords:** Insulin resistance, IRS1/PI3K/AKT/GSK3β pathway, Network Pharmacology, Non-alcoholic fatty liver disease, Traditional Chinese medicine

## Abstract

**Context:**

Jiu Wei Qing Zhi Gao (JWQZG), a traditional Chinese medicine (TCM) formulation, is widely utilized in China for managing non-alcoholic fatty liver disease (NAFLD).

**Objective:**

This study aimed to elucidate the therapeutic mechanisms of JWQZG in the management of NAFLD.

**Materials and methods:**

Network pharmacology was employed to predict the potential mechanisms of JWQZG in NAFLD management. In vivo experiments were conducted using C57BL/6J mice fed a high-fat diet (HFD) for 16 weeks, followed by treatment with JWQZG at three dosages (1.85, 3.7, and 7.4 g/kg/day) or metformin (150 mg/kg/day) for 8 weeks. In vitro studies utilized HepG2 cells exposed to 0.5 mM palmitic acid (PA) for 24 h to establish an NAFLD model, followed by exposure to JWQZG-containing serum at three concentrations for an additional 24 h. Western blot analysis was used to analyze the expression levels of key signaling pathway components.

**Results:**

Results of network pharmacology analysis identified the insulin signaling pathway as a potential mediator of the protective effects of JWQZG in NAFLD. Treatment with JWQZG markedly reduced hepatic steatosis and improved insulin resistance. This was accompanied by enhanced expression of key components in the insulin signaling pathway, including insulin receptor substrate 1 (IRS1), phosphorylated PI3K (p-PI3K), phosphorylated AKT (p-AKT), and phosphorylated GSK3β (p-GSK3β), compared to the NAFLD model group.

**Conclusions:**

These findings provide robust evidence supporting the therapeutic potential of JWQZG in NAFLD and its modulation of the insulin signaling pathway. Furthermore, the study offers valuable insights for the discovery of anti-NAFLD compounds derived from TCM formulations.

**Supplementary Information:**

The online version contains supplementary material available at 10.1186/s41065-025-00427-2.

## Introduction

Non-alcoholic fatty liver disease (NAFLD) encompasses a spectrum of liver conditions ranging from non-alcoholic fatty liver (NAFL) to non-alcoholic steatohepatitis (NASH). It is characterized by excessive triglyceride accumulation, inflammation, and hepatocyte injury. NAFLD has become the most prevalent chronic liver disease globally, with an estimated prevalence of approximately 25% [1–3]. NAFLD is bidirectionally linked to metabolic syndrome [4] and type 2 diabetes [5], while also significantly increasing the risk of cirrhosis and hepatocellular carcinoma [6]. Despite its growing prevalence, there are currently no FDA-approved pharmacological treatments for NAFLD, and lifestyle modifications remain the primary management strategy [7]. This underscores the urgent need for the development of targeted, safe, and effective medications.

According to traditional Chinese medicine (TCM), the pathogenesis of NAFLD is associated with “dampness-heat“ [8] and “spleen deficiency” [9]. Poor dietary habits such as overeating, impair spleen function and disrupt the movement of “spleen qi,” leading to the accumulation of dampness-heat and the formation of turbid phlegm, ultimately contributing to the development of NAFLD.

In recent decades, TCM formulas composed of various herbal components have demonstrated notable efficacy in the management of NAFLD. Among these, JWQZG, a novel TCM formula developed by Jiangsu Province Hospital of Chinese Medicine (Chinese Patent No. ZL202011429777.1), is recognized for its abilities to clear dampness-heat, invigorate the spleen, and supplement “qi.” This formula contains the following ingredients: *Sedum sarmentosum* Bunge (Crassulaceae) (Chuipencao), *Schisandra chinensis* (Turcz.) Baill. (Schisandraceae) (Wuweizi), *Paeonia lactiflora* Pall. (Paeoniaceae) (Baishao), *Lycopus lucidus* Turcz. ex Benth. (Lamiaceae) (Zelan), *Poria cocos* (Schw.) Wolf (Polyporaceae) (Fuling), *Coix lacryma-jobi var. ma-yuen* (Rom.Caill.) Stapf (Poaceae) (Yiyiren), *Crataegus pinnatifida* var. major (Rosaceae) (Shanzha), *Coptis chinensis* Franch. (Ranunculaceae) (Huanglian), and *Faeces Bombycis* (silkworm excrement) (Cansha).

JWQZG has been extensively used in China for several years in the treatment of NAFLD and has demonstrated significant clinical efficacy [10]. The herbal components within this formula are well-documented in the Chinese Pharmacopoeia, with some known to exert beneficial effects in managing obesity and metabolic syndrome. For instance, studies have shown that extracts of Huanglian significantly suppress the expression of C/EBP-α in adipose tissue in high-fat diet (HFD)-induced mice [11]. Additionally, flavanones derived from Chuipencao have been shown to mitigate carbon tetrachloride (CCl4)-induced liver fibrosis in rats by modulating the TGF-β1/TβR/Smad signaling pathway [12]. However, the molecular mechanisms underlying the therapeutic effects of JWQZG on NAFLD remain inadequately understood and require further elucidation.

While the exact pathogenesis of NAFLD is not fully elucidated, the widely accepted “multiple-hits” hypothesis encompasses factors such as lipotoxicity, endoplasmic reticulum (ER) stress, insulin resistance, mitochondrial dysfunction, oxidative stress, and gut microbiota dysregulation contribute to it development [13]. These complex mechanisms often limit the efficacy of single-compound treatments for NAFLD. In contrast, TCM formulas, distinguished by their multi-component and multi-target characteristics, offer potential advantages in addressing the multifaceted nature of NAFLD.

In alignment with this complexity, network pharmacology—a methodology designed to assess the effects of multi-component drugs on the human body—has emerged as a valuable tool for elucidating the molecular mechanisms underlying complicated diseases.

Accordingly, this study utilized a network pharmacology approach to investigate the therapeutic effects of JWQZG on NAFLD. To validate the findings derived from network pharmacology, a series of in vivo and in vitro experiments were conducted.

## Materials and methods

### Network Pharmacology

#### Collection of chemical compounds and prediction of potential targets

The chemical compounds present in JWQZG were identified using a network pharmacology platform. Data on these compounds, along with their absorption, distribution, metabolism, and excretion (ADME) parameters, were retrieved from the Traditional Chinese Medicine Systems Pharmacology Database and Analysis Platform (TCMSP) [14] and the Herb Database (HERB) [15]. The inclusion criteria for bioactive compounds were an oral bioavailability (OB) value of ≥ 30% (indicating systemic bioavailability following oral absorption and distribution) and a drug-likeness (DL) value of ≥ 0.18 (reflecting structural similarity to clinically used drugs listed in the DrugBank database). Subsequently, the canonical Simplified Molecular Input Line Entry System (SMILES) representations of the selected active compounds were obtained from the PubChem database [16]. These representations were submitted to the SwissTargetPrediction database to identify predicted targets, applying a target probability threshold of > 0.01 as the selection criterion [17]. 

#### Screening of gene targets for NAFLD

Disease-related genes for NAFLD were identified by querying multiple databases with the keywords “Non-alcoholic Fatty Liver Disease” and “Nonalcoholic Steatohepatitis”. The databases searched included OMIM [18], DrugBank [19], TTD [20], GeneCards [21], and DisGeNET [22]. The resulting data were then consolidated and duplicate entries were removed.

#### Compound-target network construction and analysis

The overlapping targets between the identified compounds and NAFLD-related genes were determined and designated as JWQZG-associated targets for the treatment of the disease. To visualize the results, the Draw Venn Diagram tool (https://bioinformatics.psb.ugent.be/webtools/Venn/) was used. Additionally, a compound-target interaction network was constructed using Cytoscape 3.9.1 software, depicting the connections between active compounds and their predicted targets.

#### Protein-protein interaction (PPI) network construction and key gene screening

The overlapping targets of JWQZG and NAFLD were used to construct a protein-protein interaction (PPI) network using the STRING database, with the minimum interaction threshold set to “highest confidence” (> 0.4). The network analysis results were subsequently imported into Cytoscape 3.9.1 for further processing. Key gene targets were identified by calculating the median values of three key topological parameters: Degree Centrality (DC), Betweenness Centrality (BC), and Closeness Centrality (CC). These indices were used to quantify the significance and relevance of the targets.

#### Gene ontology (GO) and Kyoto encyclopedia of genes and genomes (KEGG) pathway enrichment analysis

To examine the potential signaling pathways associated with the overlapping targets, the Metascape database [23] was used to conduct GO and KEGG pathway enrichment analyses, applying an FDR < 0.05. The GO enrichment analysis encompassed three categories: BP, MF, and CC. For further analysis, significantly enriched BPs, MFs, CCs, and KEGG pathways with a *p*-value of < 0.01 were selected. Among these, the top 10 enriched BPs, MFs, and CCs, as well as the top 15 KEGG pathways, were visualized using the Hiplot web service [24].

#### Molecular docking analysis

Molecular docking analysis was used to determine whether the key targets had good stability with the corresponding active compounds. UniProt (https://www.uniprot.org) and PubChem database (https://pubchem.ncbi.nlm.nih.gov/) were used to download the 3D structures of key targets and active compounds. Next, the AutoDockTools-1.5.7 software was used to add polar hydrogen and distribute the charge, the resulting structure was saved in “pdbqt” format. Molecular docking analysis was performed using AutoDock Vinna (v1.1.3) software. Affinity reflects the score for molecular docking. In this study, an affinity of less than − 5 kcal/mol was considered to indicate strong binding activity [25].

### Experimental verification of the protective effects of JWQZG against NAFLD

#### Drug preparation

JWQZG, was formulated using nine herbal components: Chuipencao (30 g, No.230201, Anhui Wansheng Chinese Herbal Pieces Co., Ltd.), Wuweizi (20 g, No.230201, Jingquan Chinese Herbal Pieces Co., Ltd.), Baishao (15 g, No.230201, Jingquan Chinese Herbal Pieces Co., Ltd.), Zelan (10 g, No.22102017, Anhui Xiehecheng Co., Ltd.), Fuling (15 g, No.20230201-01, Guizhou Tongde Pharmaceutical Co., Ltd.), Yiyiren (30 g, No.20230201-01, Guizhou Tongde Pharmaceutical Co., Ltd.), Cansha (10 g, No.23021306, Anhui Xiehecheng Co., Ltd.), Shanzha (10 g, No.230201, Jingquan Chinese Herbal Pieces Co., Ltd.), and Huanglian (9 g, No.20221101-01, Guizhou Tongde Pharmaceutical Co., Ltd.). The formulation was provided by the Department of Pharmacology of Jiangsu Province Hospital of Chinese Medicine. The crude herbal components from JWQZG were soaked in distilled water at a ratio of 6:1 (water volume to herb weight) for 30 min and subsequently decocted at 100 °C for 30 min. The decoction was filtered, and the procedure was repeated twice. The combined filtrates were concentrated under reduced pressure at 60 °C to yield a thick paste with a relative density of 1.30, and supplemented with sucralose and dextrin. The paste was then vacuum-dried, pulverized, and packaged, yielding a dry powder extract with a crude drug concentration of approximately 2 g/g.

Reference standards used for quality control included paeoniflorin (purity ≥ 96.8%, Batch No. 110736–202044) and quercetin (purity ≥ 98%, Batch No. 100081–201610), purchased from the National Institutes for Food and Drug Control (Beijing, China). Additionally, epiberberine (purity ≥ 98%, Batch No. J24HB186173), berberine (purity ≥ 98%, Batch No. S01A10K94340), and kaempferol (purity ≥ 98%, Batch No. A01HB190000) were obtained from Shanghai YuanYe Bio-technology Co., Ltd. (Shanghai, China).

Drug Dose Selection: The daily dosage of JWQZG for adults is 25 g. In the experimental setup, the medium dose was determined based on an equivalent conversion of the clinical dosage typically used for human adults, assuming a standard body weight of 60 kg. The medium dose for mice was calculated as 3.7 g/kg ≈ 9.1 × human (Standard weight 60 kg). The low dose was set at half of the equivalent medium dose, while the high dose was defined as twice the equivalent medium dose.

Metformin (Batch No. H20023370, Sino-American Shanghai Squibb Pharmaceuticals Ltd.) was used as a positive control at a dose of 150 mg/kg, derived from an equivalent adult dose of 1.0 g/day.

#### Ultra-performance liquid chromatography (UPLC) analysis

The homogeneity of JWQZG was assessed using UPLC. Reference solutions were prepared by accurately weighing and dissolving the target compounds in methanol. JWQZG samples were dissolved in a methanol-hydrochloric acid mixture (4:1). All working solutions were filtered through a 0.22-µm membrane filter before analysis.

UPLC analysis was conducted using an Agilent 1290 UPLC system (Agilent Technologies, Palo Alto, USA) equipped with a Poroshell 120 SB-C18 column (3.0 mm × 100 mm, 2.7 μm). The mobile phase consisted of two solvents: distilled water containing 0.1% phosphoric acid (A) and acetonitrile (B). A gradient elution was applied at a flow rate of 0.40 mL/min with the following program: 0–15 min: 10–20% B, 15–40 min: 20–30% B, 40–50 min: 30–40% B, 50–60 min: 40–50% B, 60–65 min: 50–10% B, 65–70 min: 10% B.

The injection volume was set at 4 µL, and the column temperature was maintained at 30 °C. Chromatographic peaks were identified and confirmed using mixed reference standards. Fingerprints of TCM were compared and analyzed using the Similarity Evaluation System for Chromatographic Fingerprint of Traditional Chinese Medicine (Committee of Chinese Pharmacopoeia, Version 2012).

#### Animals and experimental treatments

Eight-week-old male C57BL/6J mice (*n* = 48; Gempharmatech Co., Ltd., Jiangsu, China) were housed under specific pathogen-free conditions in the animal facility at Nanjing University of Chinese Medicine. Environmental conditions were maintained at a temperature range of 22 °C to 25 °C, a humidity level of 60%, and a 12-hour light-dark cycle, with ad libitum access to water and a standard diet. Following a one-week acclimatization period, the mice were assigned to two dietary groups. One group received a standard chow diet (control group, Con, *n* = 8), while the other group was provided a high-fat diet (HFD; 60% kcal from fat) for 16 weeks to establish a nonalcoholic fatty liver disease (NAFLD) model (*n* = 40).

At the end of the 16-week dietary period, the HFD-fed mice were randomly allocated into five treatment groups (*n* = 8 per group): JWL Group: HFD with JWQZG at a low dose (1.85 g/kg/day, intragastric administration [i.g.]), JWM Group: HFD with JWQZG at a medium dose (3.7 g/kg/day, i.g.), JWH Group: HFD with JWQZG at a high dose (7.4 g/kg/day, i.g.), Met Group: HFD with metformin (150 mg/kg/day, i.g.), HFD Group: HFD with isometric saline (i.g.).

Metformin, an insulin-sensitizing agent, was used as the positive control drug [26]. After 8 weeks of treatment, glucose tolerance tests (GTT) and insulin tolerance tests (ITT) were conducted. For the GTT, venous blood glucose was measured at baseline (0 min) and at 15, 30, 60, and 120 min following the intragastric administration of glucose (2.5 g/kg). For the ITT, blood glucose levels were similarly measured at the same time points following an intraperitoneal injection of insulin (0.75 IU/kg). Subsequently, blood and tissue samples were collected.

The study protocols were approved by the Experimental Animal Ethical Committee of Nanjing University of Chinese Medicine (Approval No. ACU220311).

#### Serum biochemical indicator determinations

Serum levels of alanine aminotransferase (ALT), aspartate aminotransferase (AST), total triglycerides (TG), total cholesterol (TC), and glucose (Glu) in mice were measured using an automatic analyzer (AU480, Beckman Coulter, Inc., USA). The assays were conducted in accordance with the instructions provided by the manufacturer.

#### Histopathological examination

Liver tissues from the six experimental groups were fixed in 10% paraformaldehyde, embedded in paraffin, and sectioned into slices measuring 3 to 5 μm in thickness. The paraffin-embedded sections were stained with hematoxylin and eosin (H&E). Frozen liver sections were stained with Oil Red O reagent to assess lipid droplet accumulation. All stained specimens were examined under a light microscope (Olympus, Tokyo, Japan).

#### Preparation of serum-containing medicine

Eight healthy male Sprague-Dawley rats, weighing 180 to 220 g, were housed under standard conditions in the animal facility at Nanjing University of Chinese Medicine. The rats were randomly assigned to four groups: the control group, JWQZG-low dose (JWL), JWQZG-medium dose (JWM), and JWQZG-high dose (JWH) groups. JWQZG was administered to the respective groups at doses of 1.85, 3.7, and 7.4 g/kg/day for 7 consecutive days, while the control group received the isometric saline.

12 h after the final gavage, blood samples were collected from the abdominal aorta of the rats. Serum was separated, incubated in a water bath at 56 °C for 30 min, filtered through a microporous membrane, and stored at -80 °C for subsequent use.

#### Cell culture and treatments

HepG2 cells, a human liver carcinoma cell line, were obtained from the Chinese Academy of Cell Resource Center (Shanghai, China) and cultured in Dulbecco’s Modified Eagle Medium (DMEM; Jiangsu Keygen Biotech Corp., Ltd.) supplemented with 10% fetal bovine serum (FBS), 100 U/mL penicillin, and 100 µg/mL streptomycin. The cells were maintained at 37 °C in a humidified atmosphere containing 5% CO_2_.

To establish an in vitro NAFLD model, HepG2 cells were treated with 0.5 mM palmitic acid (PA) for 24 h [27]. The cells were then allocated into five groups: Con Group: HepG2 cells without PA treatment, PA Group: HepG2 cells treated with PA, JWL Group: PA-treated HepG2 cells supplemented with JWQZG-low dose, JWM Group: PA-treated HepG2 cells supplemented with JWQZG-medium dose, JWH Group: PA-treated HepG2 cells supplemented with JWQZG-high dose.

For the JWQZG-treated groups, 1% serum containing either saline or varying concentrations of JWQZG was added to the respective groups. Additionally, HepG2 cells were cultured in the absence or presence of NT157 (1 µM, CAS No. 1384426-12-3; Nanjing Jingzhu Bio-technology Co., Ltd.) for 24 h to assess the specificity of JWQZG in activating IRS1 [28, 29]. 

#### CCK-8 assay

The effects of JWQZG, with or without NT157, on HepG2 cell viability were assessed using a CCK-8 assay. HepG2 cells were treated with serum containing saline or JWQZG for 24 h. Following treatment, 10 µL of CCK-8 solution was added to each well and incubated with the cells at 37 °C for 2 h. The absorbance of the reaction was measured at a wavelength of 450 nm using a microplate reader. Cell viability in the treated groups was expressed as a percentage when compared to the viability of untreated cells.

#### Hepatic TG and glycogen content assays

HepG2 cells were seeded in 96-well plates at a density of 1 × 10^4^ cells per well and cultured with palmitic acid (PA) to establish a NAFLD model. The cells were then treated with serum containing either saline or JWQZG for 24 h. Intracellular TG and glycogen levels were quantified using TG and glycogen assay kits (Nanjing Jiancheng Biotechnology Co., Ltd.) according to manufacturer’s instructions.

#### Enzyme-linked immunosorbent assay

The concentrations of cytokines, including TNF-α, IL-6, and IL-1β, in serum and cell culture supernatants were determined using ELISA kits (Guangzhou Ruixin Biotechnology Co., Ltd.). Serum insulin levels were quantified using an insulin ELISA kit (Enzyme Linked Biotechnology Co., Ltd.).

### Western blot analysis

Liver tissues or HepG2 cells were lysed in RIPA buffer, and total protein content was quantified using a BCA protein assay kit (Sparkjade, Shandong, China). Equal amounts of protein (50 µg per sample) were separated on a 10% SDS-polyacrylamide gel and subsequently transferred onto polyvinylidene difluoride (PVDF) membranes. Following transfer, the membranes were blocked with 5% non-fat dry milk for 1 h at room temperature. The membranes were then incubated overnight at 4 °C with the following primary antibodies: IRS1 (17509-1-AP, 1:1,000, Proteintech), Phospho-PI3Kp85 (4228T, 1:1,000, CST), Phospho-AKT(Ser473) (4060T, 1:1,000, CST), Phospho-GSK-3β (Ser9) (5558T, 1:1,000, CST), GSK3B (22104-1-AP, 1:1,000, Proteintech), AKT (10176-2-AP, 1:1,000, Proteintech), PI3 Kinase p85 (60225-1-Ig, 1:1,000, Proteintech), and GAPDH (10494-1-AP, 1:1,000, Proteintech) overnight. They were incubated with the secondary antibody horseradish peroxidase (HRP)-labeled goat anti-mouse or -rabbit immunoglobulin (HRP60004,1:10,000, Proteintech) at room temperature for 1 h. The blots were visualized using an enhanced chemiluminescence kit.

### Immunohistochemical staining

Paraffin-embedded liver tissue sections were deparaffinized and rehydrated, followed by antigen retrieval. The sections were then incubated overnight at 4 °C with a primary antibody against pAKT (Ser473) (p-AKT; Proteintech, 80455-1-RR, 1:200). Following primary antibody incubation, the sections were treated with a secondary antibody (HRP60004, 1:10,000, Proteintech) for 2 h at room temperature. The sections were then counterstained with hematoxylin.

### Statistical analysis

All data were expressed as the mean ± standard error of the mean (SEM). Statistical analyses were conducted using one-way analysis of variance (ANOVA). A *p* < 0.05 was considered statistically significant. Data processing and visualization were conducted using SPSS software version 20.0 and GraphPad Prism version 8.0.

## Results

### Screening of chemical compounds and potential targets of JWQZG

Through analysis using the TCMSP and HERB databases, complemented by a review of the relevant literature, 106 primary chemical compounds from the nine herbs in JWQZG were identified (Supplementary Table 1), corresponding to 956 unique targets after removing duplicates (Supplementary Table 2). Twenty-eight characteristic peaks, consistent across all three batches of JWQZG samples (Batch Nos. 2304001, 2304002, and 2304003), were identified, representing the comprehensive chemical composition of the formula. Key compounds obtained from network databases, included paeoniflorin, epiberberine, berberine, quercetin, and kaempferol (Supplementary Fig. 1).

### Collection of disease targets for NAFLD

**Fig. 1:**
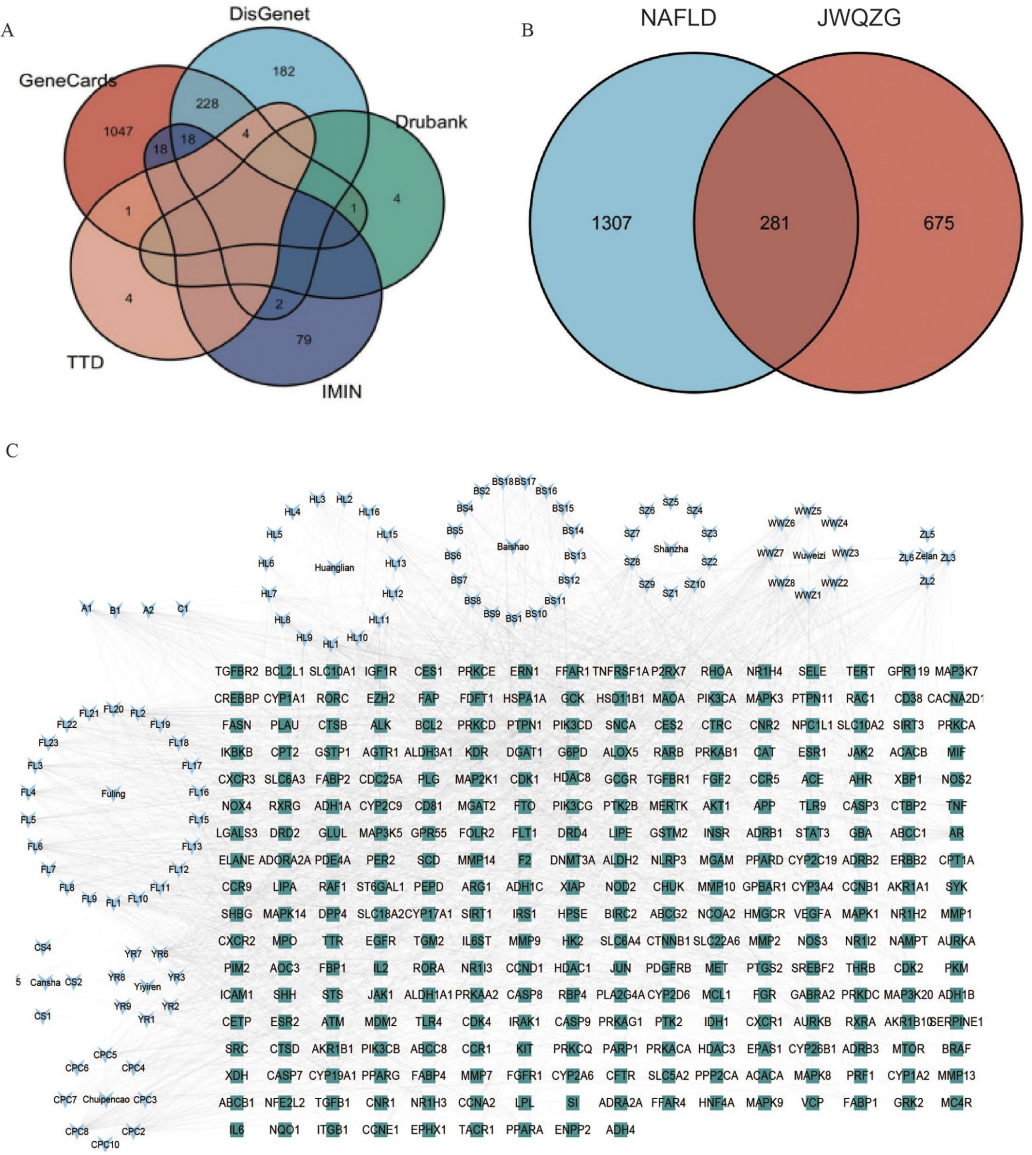
Potential targets of JWQZG against NAFLD. (**A**) Venn diagram illustrating the gene targets associated with NAFLD identified from five databases. Each color represents a specific database. (**B**) Venn diagram showing the overlap of targets between JWQZG and NAFLD. The orange section represents JWQZG-specific targets, while the blue section represents NAFLD-specific targets. (**C**) Compound-target network for JWQZG in the treatment of NAFLD. Blue nodes represent the chemical compounds in JWQZG, green nodes represent gene targets, and the edges indicate interactions between the compounds and targets. JWQZG: Jiu Wei Qing Zhi Gao

A total of 1,882 gene targets associated with NAFLD were identified from five databases: OMIM, DrugBank, TTD, GeneCards, and DisGeNET. After removing 294 duplicate entries, 1,588 unique targets were obtained (Fig. 1A and Supplementary Table 3).

### Construction of compound-target network

A total of 281 overlapping targets between the identified compounds in JWQZG and NAFLD-related targets were identified and visualized using the “Draw Venn Diagram” web service (Fig. 1B). The compound-target interaction network was constructed (Fig. 1C), comprising of 388 nodes and 2,333 edges.

### PPI network construction and key gene targets screening

**Fig. 2:**
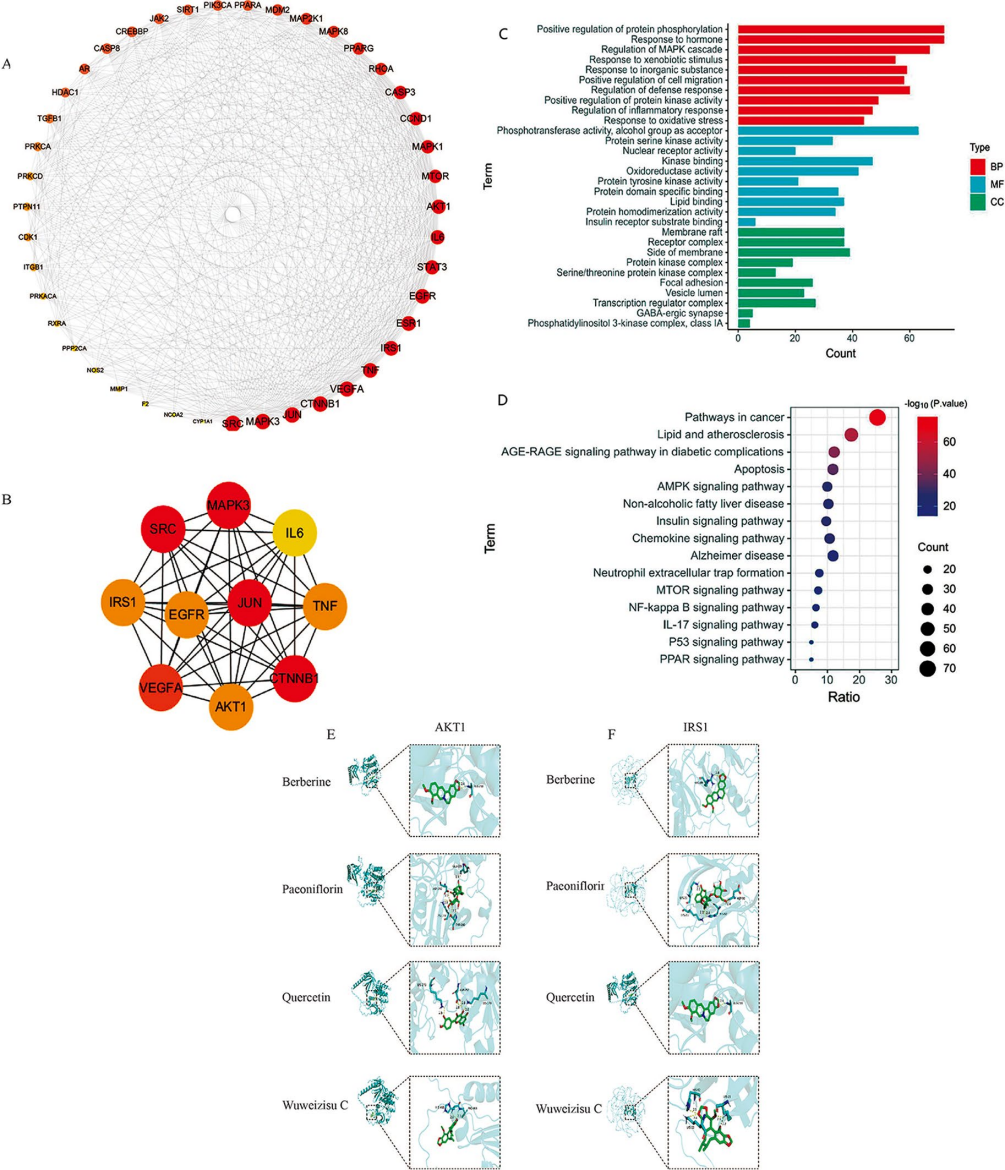
Protein-protein interaction (PPI) network analysis of coexistent targets, Gene Ontology (GO) and Kyoto Encyclopedia of Genes and Genomes (KEGG) enrichment analysis for gene targets of JWQZG in the treatment of NAFLD. (**A**) PPI network of 43 key genes identified using Degree Centrality (DC), Betweenness Centrality (BC), and Closeness Centrality (CC). (**B**) Network of the top 10 gene targets ranked by Degree values. Circle nodes represent key gene targets, with node size and color (ranging from yellow to red) reflecting their Degree values, transitioning from low to high. (**C**) GO enrichment analysis depicting the top 10 terms for BP, MF, and CC. The x-axis represents the number of enriched genes, and the y-axis lists the GO terms. (**D**) KEGG pathway enrichment analysis revealing the top 15 pathways for gene targets of JWQZG. Node size represents the count of enriched genes, while node color reflects the statistical significance (p-values). (**E**) Molecular docking of AKT1 (AF_P31749 ) with Berberine (affinity=-8.8 kcal/mol), Paeoniflorin (affinity=-8.5 kcal/mol), Quercetin (affinity=-8.4 kcal/mol), Wuweizisu C (affinity=-7.3 kcal/mol). (**f**) Molecular docking of IRS1 (AF_P35568 ) with Berberine (affinity=-6.2 kcal/mol), Paeoniflorin (affinity=-6.2 kcal/mol), Quercetin (affinity=-6.5 kcal/mol), Wuweizisu C (affinity=-6.4 kcal/mol).JWQZG: Jiu Wei Qing Zhi Gao

All 281 overlapping targets were submitted to the STRING database for PPI network analysis, and the results were imported into Cytoscape 3.9.1. A total of 43 key targets were identified, and the PPI network is depicted in Fig. 2A. Descriptions of the genes with a degree greater than 20 are summarized in Table 1, while the interaction network of the top 10 genes is depicted in Fig. 2B.

**Table 1:** The top 20 genes in PPI network

Name	Description	GeneCards ID	Betweenness Centrality	Degree
SRC	SRC Proto-Oncogene, Non-Receptor Tyrosine Kinase	GC20P037344	0.0258	41
MAPK3	Mitogen-Activated Protein Kinase 3	GC16M041633	0.0192	39
CTNNB1	Catenin Beta 1	GC03P041194	0.0191	39
JUN	Jun Proto-Oncogene, AP-1 Transcription Factor Subunit	GC01M058780	0.0192	39
VEGFA	Vascular Endothelial Growth Factor A	GC06P043770	0.0154	38
EGFR	Epidermal Growth Factor Receptor	GC07P055019	0.0148	37
TNF	Tumor Necrosis Factor	GC06P111998	0.0199	37
ESR1	Estrogen Receptor 1	GC06P151656	0.0225	37
IRS1	Insulin Receptor Substrate 1	GC02M226731	0.0128	37
AKT1	AKT Serine/Threonine Kinase 1	GC14M104769	0.0151	36
STAT3	Signal Transducer And Activator Of Transcription 3	GC17M042313	0.0108	36
IL6	Interleukin 6	GC07P022725	0.0174	36
MAPK1	Mitogen-Activated Protein Kinase 1	GC22M021759	0.0111	34
CCND1	Cyclin D1	GC11P069641	0.0074	34
CASP3	Caspase 3	GC04M184627	0.0087	34
MTOR	Mechanistic Target Of Rapamycin Kinase	GC01M011106	0.0073	34
PPARG	Peroxisome Proliferator Activated Receptor Gamma	GC03P012287	0.0131	31
RHOA	Ras Homolog Family Member A	GC03M049359	0.0069	31
MAP2K1	Mitogen-Activated Protein Kinase Kinase 1	GC15P066386	0.0067	30
MAPK8	Mitogen-Activated Protein Kinase 8	GC10P048306	0.0064	30
MDM2	MDM2 Proto-Oncogene	GC12P068808	0.0033	29
SIRT1	Sirtuin 1	GC10P067884	0.0046	27
CREBBP	CREB Binding Protein	GC16M013493	0.0081	27
PIK3CA	Phosphatidylinositol-4,5-Bisphosphate 3-Kinase Catalytic Subunit α	GC03P179148	0.0032	27
CASP8	Androgen Recepto	GC0XP067544	0.0017	27
AR	Caspase 8	GC02P201233	0.008	27
PPARA	Peroxisome Proliferator Activated Receptor α	GC22P046150	0.0121	27
JAK2	Janus Kinase 2	GC09P004985	0.0041	27
HDAC1	Histone Deacetylase 1	GC01P032292	0.0045	25
TGFB1	Transforming Growth Factor Beta 1	GC19M041301	0.0035	24
PRKCA	Protein Kinase C Alpha	GC17P066302	0.0034	23
PTPN11	Protein Tyrosine Phosphatase Non-Receptor Type 11	GC12P112418	0.0013	21
CDK1	Cyclin Dependent Kinase 1	GC10P060772	0.0026	21
PRKCD	Protein Kinase C Delta	GC03P053156	0.0012	21
ITGB1	Integrin Subunit Beta 1	GC10M034762	0.0011	20
PRKACA	Protein Kinase CAMP-Activated Catalytic Subunit α	GC19M015906	0.0016	18
RXRA	Retinoid X Receptor Alpha	GC09P134317	0.005	17
PPP2CA	Protein Phosphatase 2 Catalytic Subunit Alpha	GC05M134194	0.0009	16
MMP1	Matrix Metallopeptidase 1	GC11M114281	0.0001	15
NOS2	Nitric Oxide Synthase 2	GC17M027756	0.0009	15
F2	Coagulation Factor II, Thrombin	GC11P047030	0.0004	13
NCOA2	Nuclear Receptor Coactivator 2	GC08M070109	0.0012	12
CYP1A1	Cytochrome P450 Family 1 Subfamily A Member 1	GC15M074719	0.0007	9

Among these key targets, several, including catenin beta 1 (CTNNB1), vascular endothelial growth factor A (VEGFA), mitogen-activated protein kinase 3 (MAPK3), insulin receptor substrate 1 (IRS1), protein kinase B (AKT1), TNF, and IL-6, have been involved in multiple biological processes associated with NAFLD, such as inflammation, insulin secretion regulation, and glucose and lipid metabolism. These findings propose that JWQZG may exert therapeutic effects on NAFLD by regulating these key targets.

### GO and KEGG enrichment analysis

The biological functions associated with JWQZG in the treatment of NAFLD were evaluated through GO enrichment analysis, which was performed on the 281 overlapping targets. The top 10 items in the categories of BP, CC, and MF are presented in Fig. 2C. Key enriched biological processes included positive regulation of protein phosphorylation (GO:0001934) and response to hormone (GO:0009725).

Subsequently, KEGG pathway enrichment analysis was conducted to identify potential pathways involved in the effects of JWQZG on NAFLD. The top 15 pathways are displayed in Fig. 2D, with significant enrichment observed in pathways such as lipid and atherosclerosis (hsa05417), AGE-RAGE signaling pathway in diabetic complications (hsa04933), NAFLD (hsa04932), and insulin signaling pathway (hsa04910).

These results collectively indicate that the therapeutic effects of JWQZG on NAFLD may be mediated through a multitarget and multi-pathway mechanism, involving processes such as lipid metabolism, insulin signaling, and inflammation.

### Docking stimulation verification

To validate the binding ability of the bioactive compounds (berberine, paeoniflorin, quercetin, and Wuweizisu C) to the key targets (IRS1, AKT1), molecular docking was performed. As shown in Figs. 2E-F and 8 pairs of affinity results were all less than − 5 kcal/mol, indicating that the four bioactive compounds exhibited a strong binding activity to IRS1 and AKT1.

### JWQZG ameliorates hepatic steatosis and inflammation in NAFLD model mice

**Fig. 3:**
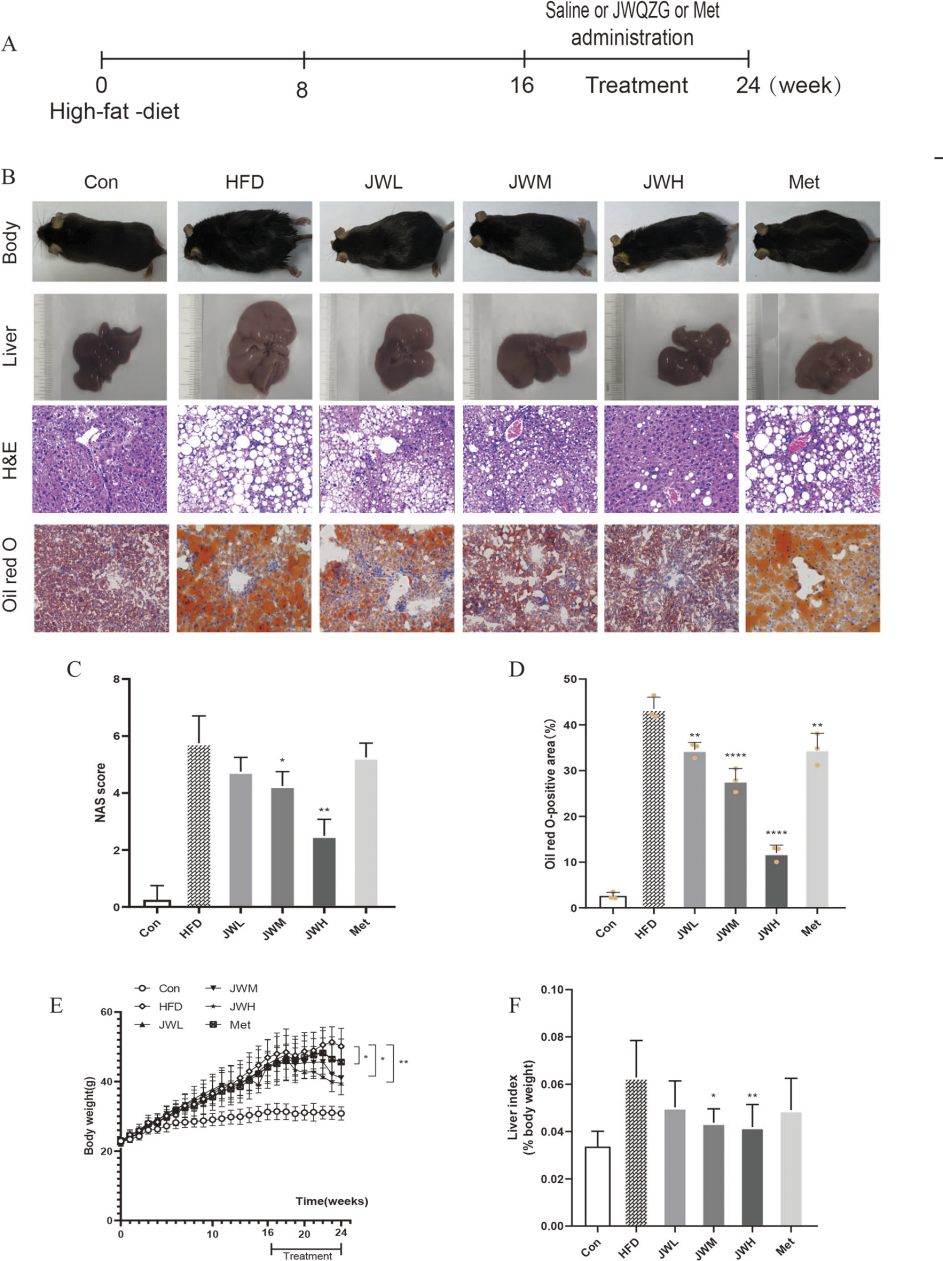
JWQZG alleviates liver injury in NAFLD mice. (**A**) Timeline of experimental modeling and drug administration. (**B**) Representative images of body and liver appearance, and liver tissue sections stained with hematoxylin and eosin (H&E) and Oil Red O (magnification, 20×) for each group. (**C**) NAFLD activity scores (NAS) in each group (*n* = 4).(**D**) Quantitative analysis of Oil Red O-stained areas in liver sections for each group (*n* = 3). (E-F) Body weight and liver index (liver-to-body weight ratio) of each group (*n* = 8). Data are presented as mean ± SD. *p* < 0.05, *p* < 0.01, *p < 0.001, **p < 0.0001 compared to the HFD group. JWQZG, Jiu Wei Qing Zhi Gao; Con, normal control (mice fed a standard chow diet and treated with saline); HFD, high-fat diet control (mice fed a high-fat diet and treated with saline); JWL, mice fed a high-fat diet and treated with low-dose JWQZG; JWM, mice fed a high-fat diet and treated with medium-dose JWQZG; JWH, mice fed a high-fat diet and treated with high-dose JWQZG; Met, mice fed a high-fat diet and treated with metformin

To assess the therapeutic effects of JWQZG on NAFLD, mice were treated with different doses of JWQZG, metformin, or saline for 8 weeks (Fig. 3). When compared to the Con group, mice in the HFD group exhibited visibly whiter livers, while those treated with metformin or varying doses of JWQZG exhibited a progressive reddening of the liver (Fig. 3B).

Treatment with medium and high doses of JWQZG significantly reduced the body weight and liver index (liver-to-body weight ratio) increases iduced by the HFD (Fig. 3E and F). Histological analysis demonstrated marked hepatocyte swelling, steatosis, and inflammatory cell infiltration in the livers of mice in the HFD group, as shown by H&E staining. Large lipid droplets were observed in the HFD group through Oil Red O staining. In contrast, treatment with JWQZG mitigated the hepatic steatosis and inflammation induced by the HFD (Fig. 3B).

The nonalcoholic fatty liver disease activity scores (NAS) were significantly reduced in the JWM and JWH JWQZG groups compared to the HFD group (Fig. 3C). Additionally, the Oil Red O positive area, indicative of lipid accumulation, was substantially reduced in the Met, JWL, JWM, and JWH groups (Fig. 3D).

These findings indicate that JWQZG effectively alleviates hepatic steatosis and inflammation in a dose-dependent manner.

### JWQZG improves serum biochemical indicators and cytokine levels in NAFLD model mice

**Fig. 4:**
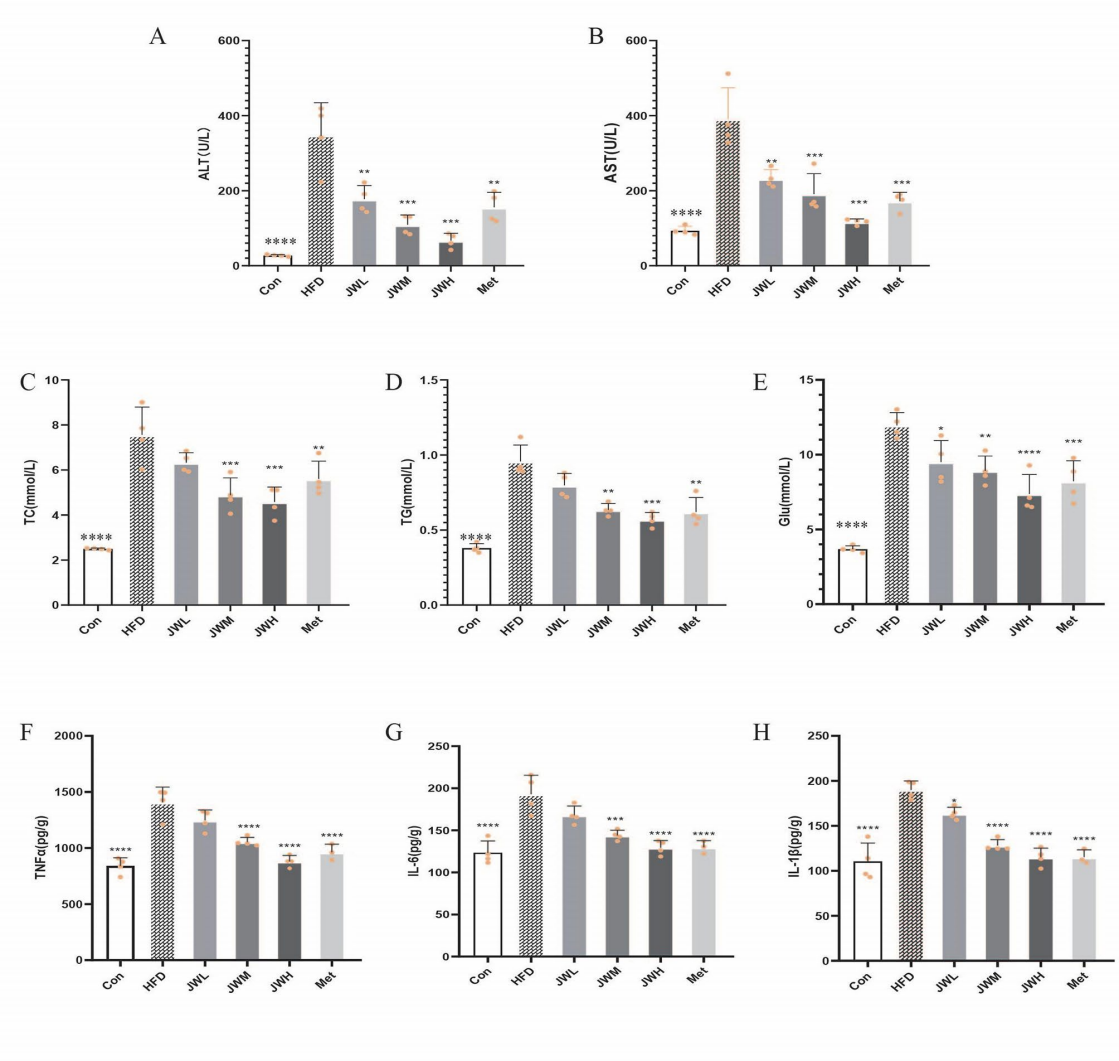
JWQZG improves serum biochemical indicators and cytokine levels in NAFLD mice. (**A**-**E**) Levels of serum ALT, AST, TC, TG, and Glu in each group (*n* = 4). (**F**-**H**) Levels of proinflammatory cytokines TNF-α, IL-6, and IL-1β in serum from each group (*n* = 4). Data are presented as mean ± SD. *p* < 0.05, *p* < 0.01, *p < 0.001, **p < 0.0001 compared to the HFD group. JWQZG, Jiu Wei Qing Zhi Gao; Con, normal control (mice fed a standard chow diet and treated with saline); HFD, high-fat diet control (mice fed a high-fat diet and treated with saline); JWL, mice fed a high-fat diet and treated with low-dose JWQZG; JWM, mice fed a high-fat diet and treated with medium-dose JWQZG; JWH, mice fed a high-fat diet and treated with high-dose JWQZG; Met, mice fed a high-fat diet and treated with metformin

The effects of JWQZG on serum biochemical indicators and cytokine levels were assessed by measuring ALT, AST, TG, TC, Glu, TNF-α, IL-6, and IL-1β. Compared to the HFD group, significant reductions in serum ALT, AST, TG, TC, and Glu levels were observed in the Met group and all JWQZG-treated groups, with the most pronounced effects seen in the JWH group (Figs. 4A–E).

Additionally, serum levels of cytokines TNF-α, IL-6, and IL-1β, which are associated with Kupffer cell activation in NAFLD, were markedly inhibited in the JWQZG-treated groups in a dose-dependent manner (Figs. 4F–H). These findings align with the results of the PPI network analysis (Fig. 2D).

Overall, these results indicate that JWQZG effectively improves liver function, regulates glycolipid metabolism, and suppresses inflammation in HFD-induced NAFLD mice.

### JWQZG alleviates insulin resistance in HFD-induced NAFLD model mice

KEGG pathway analysis indicated that the insulin signaling pathway may play a key role in the therapeutic effects of JWQZG on NAFLD. To assess the impact of JWQZG on insulin resistance, GTT, ITT, and the homeostatic model assessment of insulin resistance (HOMA-IR) were tested.

**Fig. 5:**
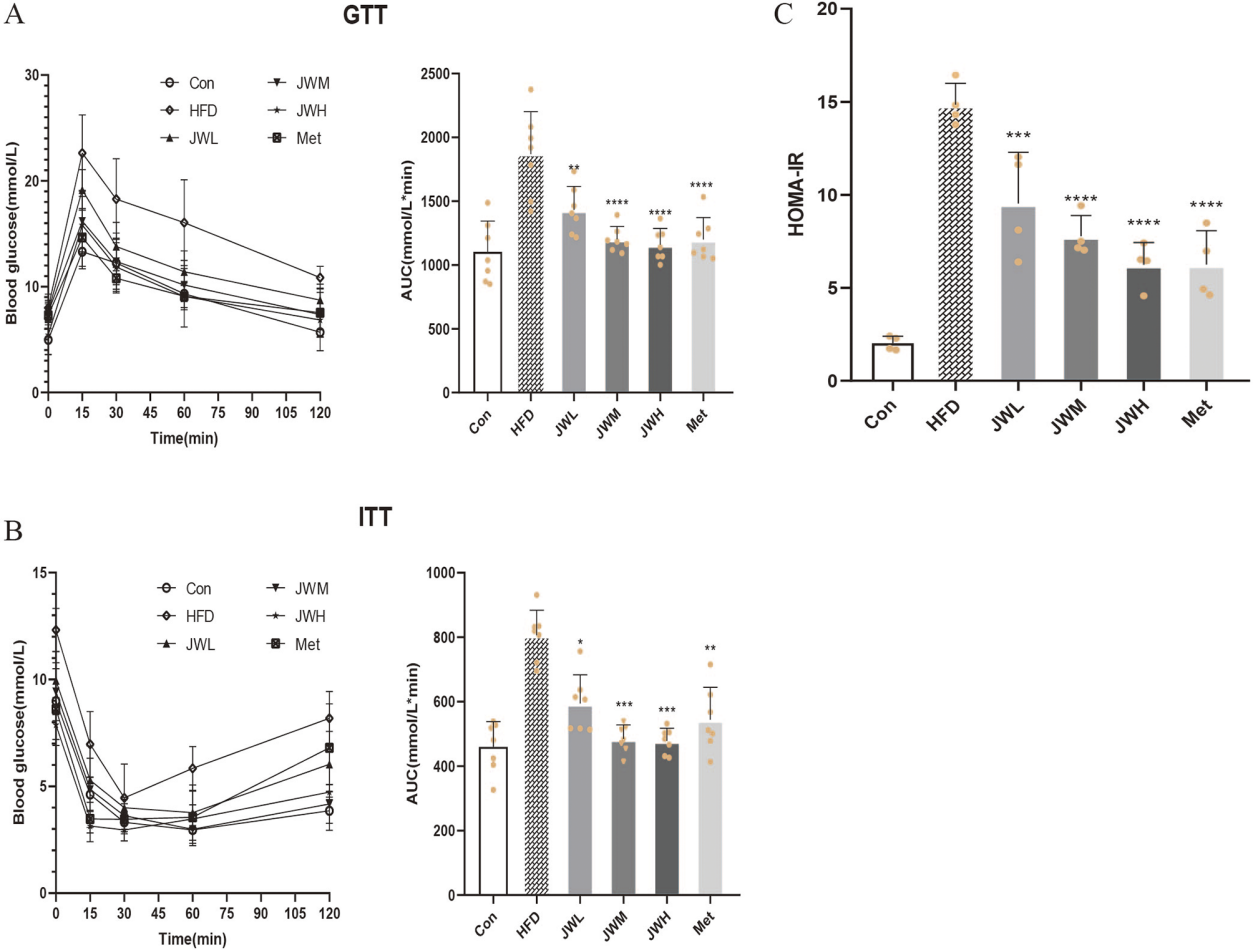
JWQZG attenuates insulin resistance in NAFLD mice. (**A**) Area under the curve (AUC) for glucose tolerance test (GTT) (*n* = 7). (**B**) AUC for insulin tolerance test (ITT) (*n* = 7). (**C**) Homeostatic model assessment of insulin resistance (HOMA-IR) values (*n* = 4). Data are presented as mean ± SD. *p* < 0.05, *p* < 0.01, *p < 0.001, **p < 0.0001 compared to the HFD group. JWQZG, Jiu Wei Qing Zhi Gao; Con, normal control (mice fed a standard chow diet and treated with saline); HFD, high-fat diet control (mice fed a high-fat diet and treated with saline); JWL, mice fed a high-fat diet and treated with low-dose JWQZG; JWM, mice fed a high-fat diet and treated with medium-dose JWQZG; JWH, mice fed a high-fat diet and treated with high-dose JWQZG; Met, mice fed a high-fat diet and treated with metformin; GTT, glucose tolerance test; ITT, insulin tolerance test; HOMA-IR, homeostatic model assessment of insulin resistance

Mice in the HFD group exhibited impaired glucose tolerance and spontaneous development of insulin resistance. In contrast, treatment with JWQZG or metformin significantly improved glucose tolerance, as demonstrated by a reduction in the area under the curve (AUC) (Fig. 5A) and increased insulin sensitivity (Fig. 5B). Moreover, both treatments substantially decreased HOMA-IR levels (Fig. 5C).

These findings indicate that JWQZG effectively mitigates insulin resistance, a key pathogenic factor in NAFLD.

### 10 JWQZG inhibits lipid accumulation and increases glycogen content in PA-induced NAFLD model cells

**Fig. 6:**
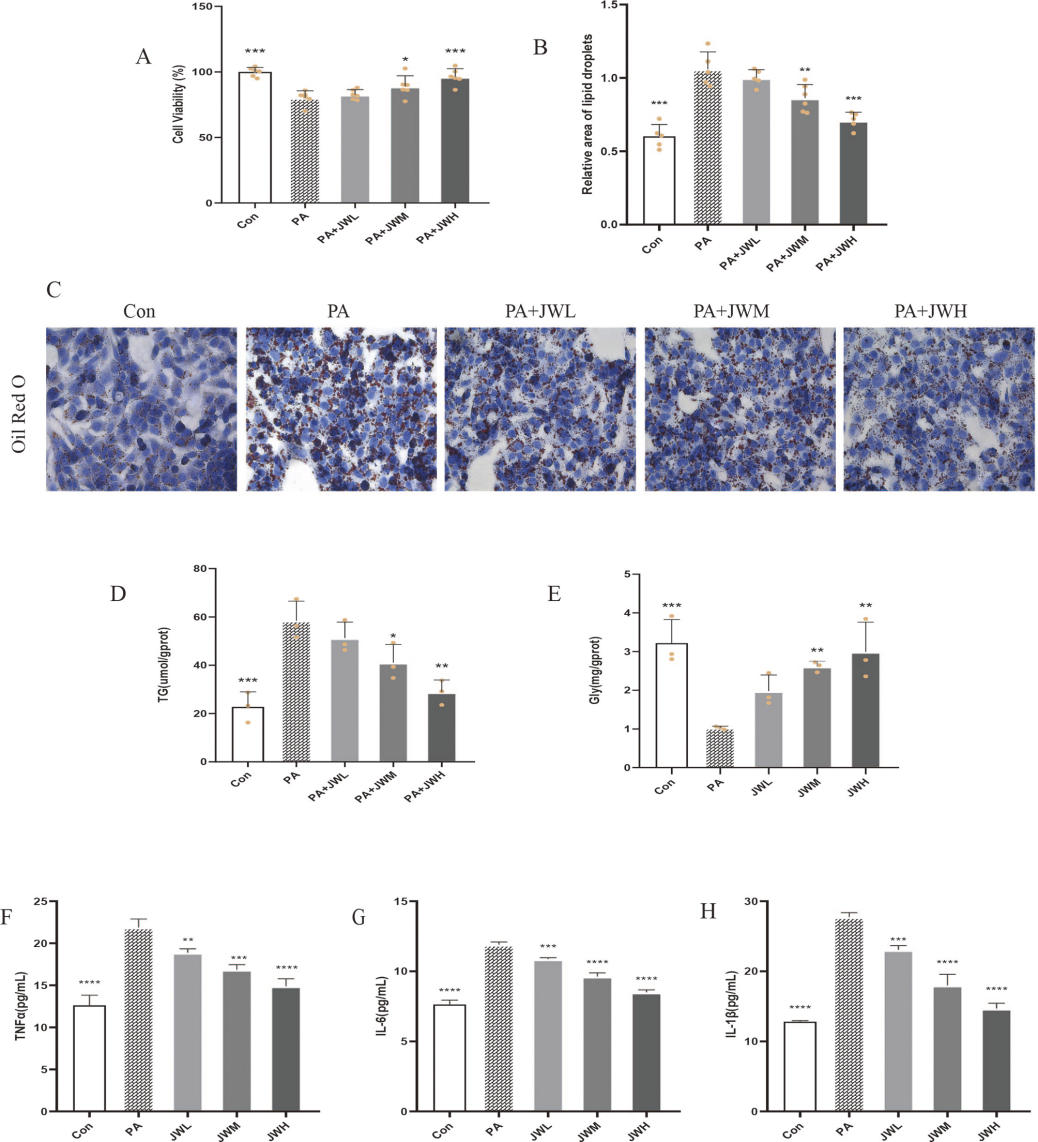
JWQZG reduces lipid accumulation in NAFLD model cells. (**A**) Viability of HepG2 cells treated with different concentrations of JWQZG-containing serum (*n* = 5). (**B**) Relative lipid area in HepG2 cells (*n* = 5). (**C**) Representative images of Oil Red O staining in HepG2 cells (magnification, 40×). (**D**-**E**) Intracellular triglyceride (TG) and glycogen (Gly) levels in HepG2 cells (*n* = 3). (**F**-**H**) Levels of TNF-α, IL-6, and IL-1β in cell culture supernatants (*n* = 3). Data are presented as mean ± SD. *p* < 0.05, *p* < 0.01, *p < 0.001, **p < 0.0001 compared to the PA group. JWQZG, Jiu Wei Qing Zhi Gao; Con, normal control (HepG2 cells incubated normally); PA, model control (HepG2 cells incubated with palmitic acid to induce the NAFLD model); JWL, NAFLD cells treated with low-dose JWQZG; JWM, NAFLD cells treated with medium-dose JWQZG; JWH, NAFLD cells treated with high-dose JWQZG; TG, triglyceride; Gly, glycogen

Cell viability was assessed using the CCK-8 assay to evaluate the effect of JWQZG. Treatment with 0.5 mM PA resulted in a significant reduction in cell viability, although no severe toxicity was observed. However, treatment with JWQZG significantly improved the viability of PA-induced HepG2 cells in a dose-dependent manner (Fig. 6A).

Oil Red O staining and TG analysis revealed that lipid accumulation in HepG2 cells was significantly suppressed in the JWQZG-treated groups compared to the PA group (Figs. 6B–D). Furthermore, intracellular glycogen content increased in a dose-dependent manner following JWQZG treatment (Fig. 6E).

The levels of proinflammatory cytokines TNF-α, IL-6, and IL-1β in the cell culture supernatants were significantly reduced in JWQZG-treated groups (Figs. 6F–H).

These findings collectively demonstrate that JWQZG effectively improves key characteristics of the NAFLD phenotype in vitro.

### 11 JWQZG activates the IRS1/PI3K/AKT/GSK3β pathway in vivo and in vitro

Based on network analysis of the top 10 genes (Fig. 2D), it was hypothesized that JWQZG may alleviate insulin resistance by modulating the expression of IRS1 and AKT. To test this hypothesis, western blot analysis was conducted to measure the expression of key proteins, including IRS1, phosphoinositide 3-kinase (PI3K), phosphorylated PI3K (p-PI3K), AKT, p-AKT, glycogen synthase kinase 3 beta (GSK3β), and phosphorylated GSK3β (p-GSK3β).

**Fig. 7:**
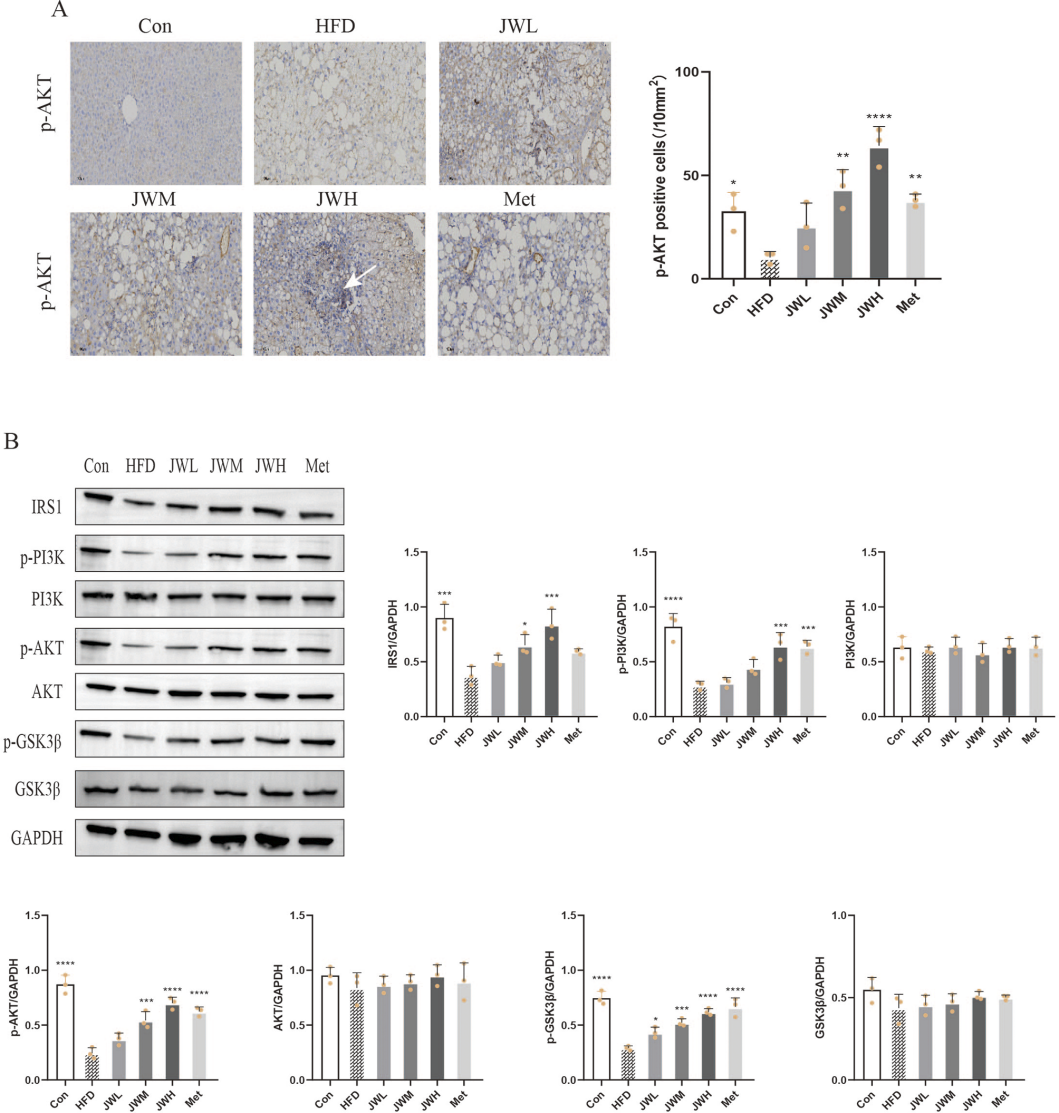
JWQZG activates the IRS1/PI3K/AKT/GSK3β pathway in NAFLD mice. (**A**) Immunohistochemical staining and quantitative analysis of p-AKT expression in liver tissue (magnification, 20×) (*n* = 3). (**B**) Relative protein expression levels of IRS1, p-PI3K, PI3K, p-AKT, AKT, p-GSK3β, and GSK3β in liver tissues measured by western blot analysis (*n* = 3). Data are presented as mean ± SD. *p* < 0.05, *p* < 0.01, *p < 0.001, **p < 0.0001 compared to the HFD group. JWQZG, Jiu Wei Qing Zhi Gao; Con, normal control (mice fed a standard chow diet and treated with saline); HFD, high-fat diet control (mice fed a high-fat diet and treated with saline); JWL, mice fed a high-fat diet and treated with low-dose JWQZG; JWM, mice fed a high-fat diet and treated with medium-dose JWQZG; JWH, mice fed a high-fat diet and treated with high-dose JWQZG; Met, mice fed a high-fat diet and treated with metformin

**Fig. 8:**
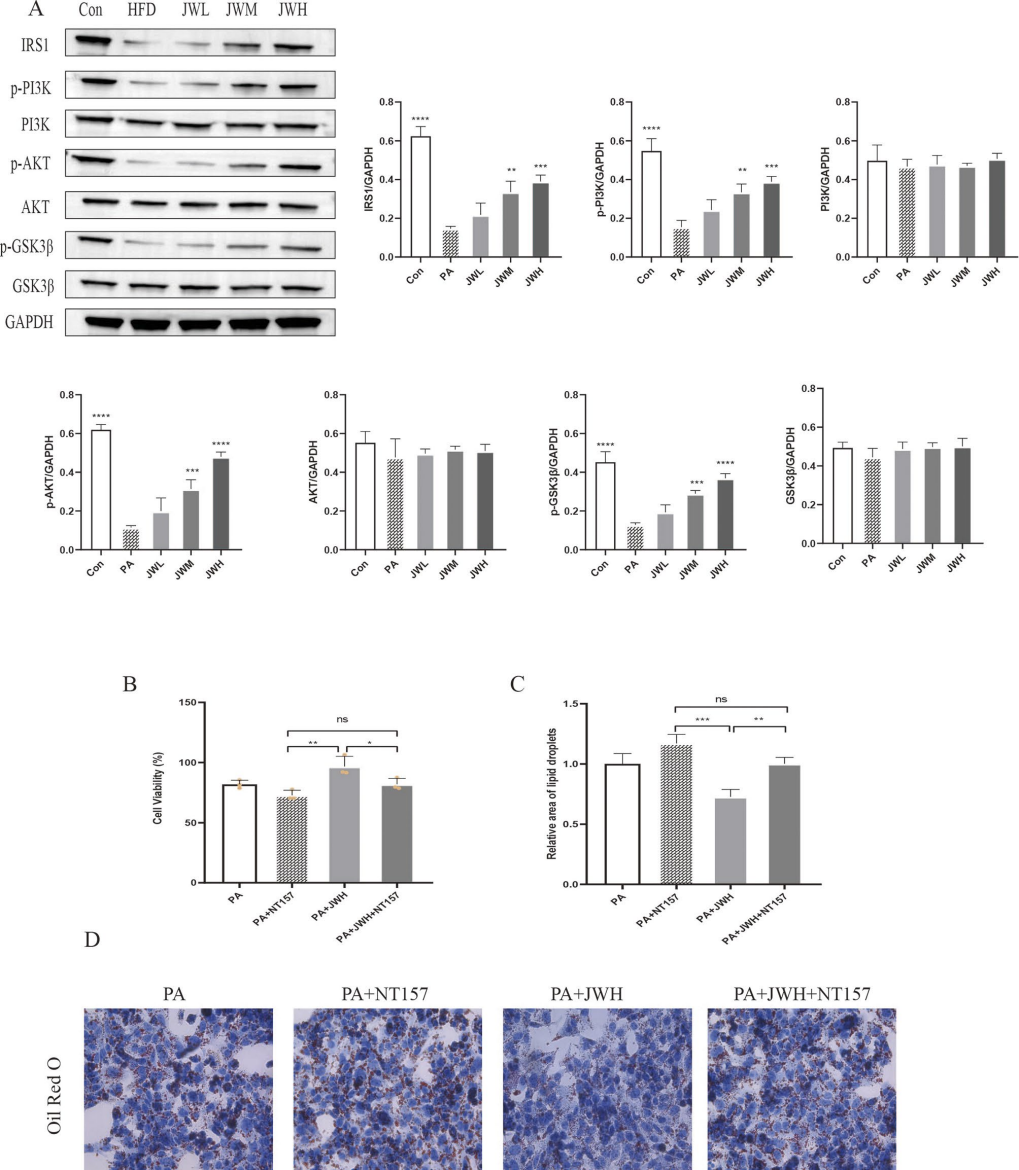
JWQZG activates the IRS1/PI3K/AKT/GSK3β pathway in NAFLD model cells. (**A**) Relative protein expression levels of IRS1, p-PI3K, PI3K, p-AKT, AKT, p-GSK3β, and GSK3β in HepG2 cells measured by western blot analysis (*n* = 3). (**B**) Viability of HepG2 cells treated with JWQZG and/or the IRS1 inhibitor NT157 (*n* = 3). (**C**) Relative lipid droplet area in HepG2 cells (*n* = 3). (**D**) Representative images of Oil Red O staining in HepG2 cells (magnification, 40×). Data are presented as mean ± SD. *p* < 0.05, *p* < 0.01, *p < 0.001, **p < 0.0001; “ns” indicates no significance. JWQZG: Jiu Wei Qing Zhi Gao; Con: normal control (HepG2 cells incubated under normal conditions); PA: model control (HepG2 cells incubated with palmitic acid to induce the NAFLD model); JWL: NAFLD cells treated with low-dose JWQZG; JWM, NAFLD cells treated with medium-dose JWQZG; JWH: NAFLD cells treated with high-dose JWQZG; PA + NT157: NAFLD cells treated with NT157; PA + JWH: NAFLD cells treated with high-dose JWQZG; PA + JWH + NT157: NAFLD cells treated with high-dose JWQZG and NT157

Immunohistochemical analysis of liver sections showed a higher number of p-AKT-positive cells in the JWH group compared to the HFD group (Fig. 7A). Additionally, western blot results demonstrated that JWQZG treatment significantly upregulated the expression of IRS1 and the phosphorylated forms of PI3K, AKT, and GSK3β in both HFD-induced NAFLD mouse livers and PA-induced NAFLD HepG2 cells. However, the total expressions of PI3K, AKT, and GSK3β remained unchanged (Figs. 7B and 8A).

These findings indicate that JWQZG mitigates liver injury and insulin resistance by activating the IRS1/PI3K/AKT/GSK3β signaling pathway.

To further investigate the mechanism underlying the effects of JWQZG, NT157, an inhibitor of IRS1, was used in PA-induced NAFLD model cells. Following NT157 treatment, the results of the CCK-8 assay and Oil Red O staining indicated that JWQZG was unable to restore cell viability or suppress lipid accumulation (Figs. 8B–D).

These findings indicate that NT157 effectively negates the protective effects of JWQZG on NAFLD, providing strong evidence that JWQZG exerts its therapeutic actions through the IRS1/PI3K/AKT/GSK3β signaling pathway.

## Discussion

The multifactorial etiology of NAFLD, involving genetic predispositions [30, 31], metabolic disturbances [32], and environmental factors [33], contributes to the complexity of its management. Genetic variants in genes such as transmembrane six superfamily member 2 (TM6SF2) [34], glucokinase regulatory protein (GCKR) [35], and patatin-like phospholipase domain-containing-3 (PNPLA3) [36] are found to associate with NAFLD and NASH, with PNPLA3 classified as one of the most common genetic variations.Patients who have the PNPLA3 genetic polymorphism produce a truncated lipase enzyme which impedes triglyceride breakdown and subsequently reduces liver triglyceride (TG) secretion in the form of very-low-density lipoproteins (VLDL). Furthermore, the microbiota is involved in alternating the balance between pro-inflammatory or anti‐inflammatory signals, which contributing to the progression in NASH. When compared to healthy controls, there is a relative abundance of potential pathogens, such as Gram-negative Proteobacteria, Enterobacteriaceae, and *Escherichia* spp. among patients with NASH, while *Faecalibacterium prausnitzii* and *Akkermansia muciniphila* are relatively diminished [37]. Current treatment strategies emphasize multimodal interventions such as lifestyle modifications, weight reduction, and the use of potential therapeutic agents. TCM has revealed significant efficacy in managing NAFLD, particularly in Asia. For instance, the Si Miao formula, a well-known TCM decoction, has been demonstrated to inhibit hepatic fatty acid synthesis and mitigate inflammation associated with NAFLD [38]. 

Experimental findings from this study indicate that JWQZG effectively reduces hepatic lipid deposition, normalizes serum lipid metabolism, and downregulates hepatic inflammatory cytokine expression. Previous studies have highlighted that patients with dampness-heat syndrome are prone to metabolic abnormalities and insulin resistance [39]. A cross-sectional analysis involving 1,677 participants reported that most of the patients with phlegm-dampness syndrome often exhibited mixed constitutions of dampness-heat or qi deficiency, with correlation coefficients of 0.42 and 0.20, respectively [40]. 

In TCM theory, JWQZG exerts its therapeutic effects through actions aimed at clearing dampness-heat, invigorating the spleen, and supplementing qi. The formula comprises the following components: Chuipencao and Huanglian designated as Jun (monarch) drugs, which primarily clear dampness-heat from the middle jiao (middle energizer). Fuling and Yiyiren, classified as Chen (minister) drugs, strengthen the spleen, replenish qi, and facilitate the removal of dampness-heat. Wuweizi and Baishao nourish yin and soften the liver, mitigating the potential excessive clearing effects of Chuipencao and Huanglian. Zelan and Shanzha, categorized as Zuo drugs, promote qi flow, enhance blood circulation, and help dispel turbidity and fat. Cansha, identified as a Shi (envoy) drug, harmonizes the stomach and guides the formula to the meridians, enhancing its overall efficacy. These components work synergistically to target the multifaceted pathophysiology of NAFLD. The clinical trial results [10] provide strong evidence supporting the therapeutic potential of JWQZG in NAFLD patients. These findings, combined with our in vivo and in vitro experimental data, suggest that JWQZG modulates the IRS1/PI3K/AKT/GSK3β signaling pathway, leading to improved liver function and reduced liver fat content. Future studies should focus on exploring the upstream regulatory factors of this pathway to further elucidate the mechanism of action of JWQZG. Given its demonstrated clinical efficacy, further investigation into the active compounds and molecular mechanisms underlying the therapeutic effects against NAFLD is warranted.

Network pharmacology analysis identified 106 active compounds and 281 potential targets associated with JWQZG. Among these compounds, quercetin and berberine have demonstrated significant experimental efficacy against NAFLD in recent years. For example, Porras et al. [41] reported that quercetin could restore gut microbiota balance and suppress endotoxemia-mediated activation of the TLR4/NF-κB pathway, subsequently inhibiting inflammasome activation and endoplasmic reticulum stress. Similarly, Wang et al. [42] found that berberine promoted SIRT1-mediated deacetylation of CPT1A at the Lys675 site, reducing ubiquitin-dependent degradation of CPT1A and ameliorating NASH.

The identified targets were associated with glycolipid metabolism-related pathways, including lipid and atherosclerosis, the AGE-RAGE signaling pathway in diabetic complications, and the insulin signaling pathway. Among the top 10 hub genes identified in the PPI network, IRS1 and AKT play key roles in these pathways.

IRS1 is essential for mediating the regulation of various cellular processes, particularly hepatic insulin signaling in insulin, which is vital for glucose homeostasis [43, 44]. When insulin binds to its receptor, autophosphorylation of the intracellular tyrosine residues of the receptor facilitates the docking and phosphorylation of insulin receptor substrates, such as IRS1/IRS2. This process activates downstream kinase cascades, including the PI3K/AKT pathway [45]. Activated AKT, in turn, phosphorylates GSK3β, inhibiting glycogen synthase and contributing to the regulation of blood glucose levels [46]. However, this pathway is often disrupted in NAFLD [47, 48].

In the present study, treatment with JWQZG significantly upregulated IRS1 expression and increased the phosphorylation of PI3K, AKT, and GSK3β in both in vitro and in vivo models. Conversely, inhibition of IRS1 with NT157 almost completely negated the protective effects of JWQZG against hepatic steatosis. These findings indicate that JWQZG improves insulin resistance in NAFLD by activating the IRS1/PI3K/AKT/GSK3β signaling pathway.

This study has several limitations that warrant consideration. First, the effects and mechanisms of JWQZG on insulin resistance require validation in a larger clinical sample to ensure robustness and generalizability. Second, the network pharmacology analysis identified numerous compounds and potential targets associated with inflammation and cell death, which present opportunities for further investigation to elucidate additional mechanisms of action.

Despite these limitations, this research provides valuable evidence supporting the clinical application of JWQZG and lays a foundation for future studies aimed at exploring its mechanisms for treating NAFLD.

## Conclusions

The therapeutic effects and mechanisms of JWQZG in the treatment of NAFLD were investigated using a combination of network pharmacology and experimental validation. The findings demonstrated that JWQZG effectively reduces hepatic lipid accumulation and inflammation while improving insulin resistance through activation of the IRS1/PI3K/AKT/GSK3β pathway.

These results highlight the value of integrating modern bioinformatics approaches with traditional experimental techniques as a robust and reliable strategy for exploring the therapeutic potential of herbal medicines.

## Electronic supplementary material

Below is the link to the electronic supplementary material.

Supplementary Material 1

Supplementary Material 2

## Data Availability

All supporting data are included within the main article.

## Abbreviations

TCM: Traditional Chinese medicine
NAFLD: Nonalcoholic fatty liver disease
JWQZG: Jiu Wei Qing Zhi Gao
Con: Control
HFD: High-fat-diet
JWL: JWQZG-low dose
JWM: JWQZG-medium dose
JWH: JWQZG-high dose
Met: Metformin
PPI: Protein-protein interaction
GO: Gene ontology
KEGG: Kyoto encyclopedia of genes and genomes
BP: Biological process
MF: Molecular function
CC: Cellular component
IRS1: Insulin receptor substrate 1
PI3K: Phosphoinositide 3-kinase
AKT: Protein kinase B
GSK3β: Glycogen synthase kinase 3 beta
AST: Aspartate aminotransferase
ALT: Glutamic pyruvic transaminase
TC: Total cholesterol
TG: Total triglyceride
LDL-C: Low-density lipoprotein cholesterol
Glu: Glucose
Gly: Glycogen
TNFα: Tumor necrosis factorα
IL-6: Interleukin 6
IL-1β: Interleukin 1β
