# Supplementary figures and images for: Mechanistic evaluation of Jiu Wei Qing Zhi Gao in non-alcoholic fatty liver disease: insights from network Pharmacology and experimental validation
Source: Hereditas. 2025 Apr 12;162:59. doi: 10.1186/s41065-025-00427-2 (PMC11992867; doi:10.1186/s41065-025-00427-2)

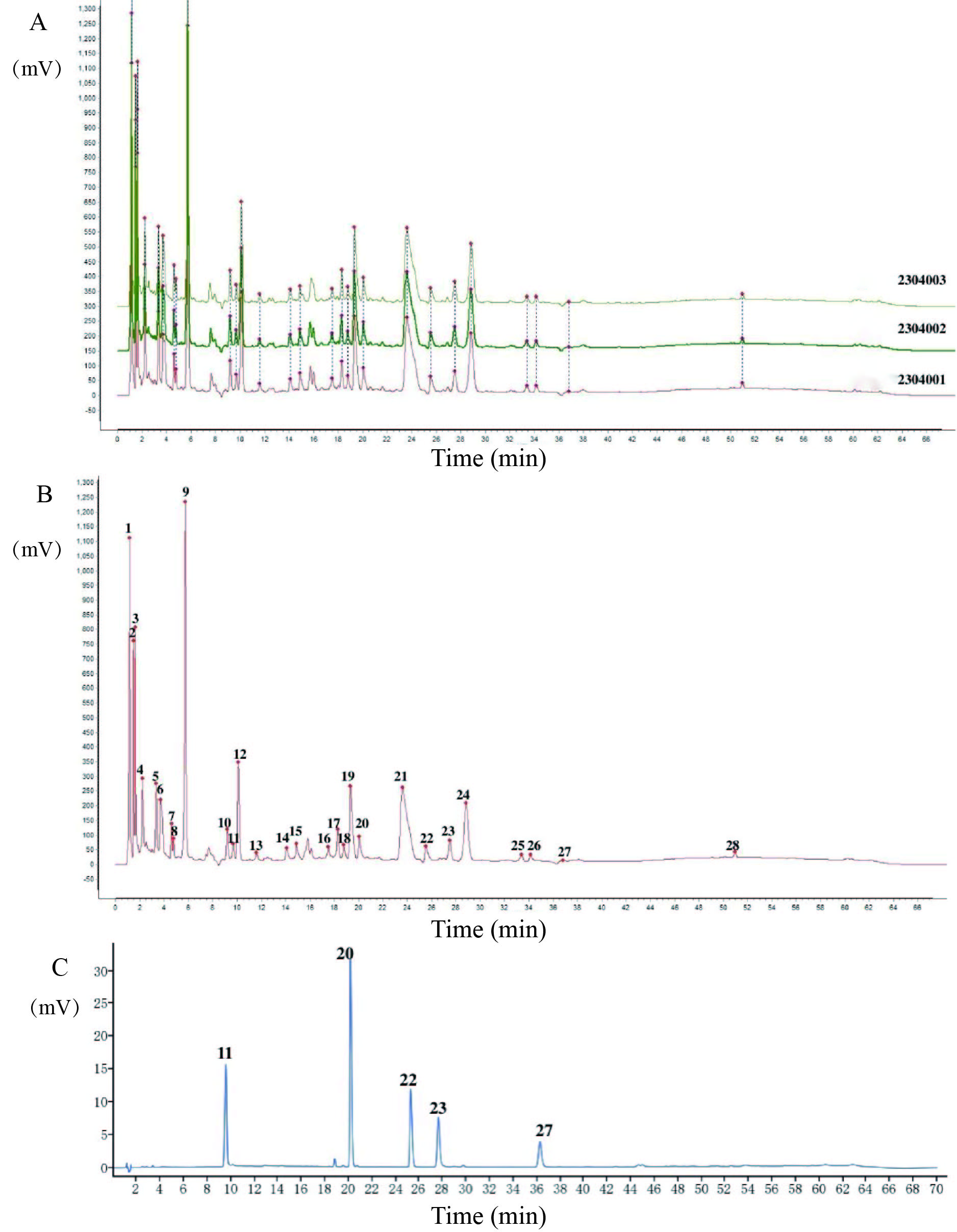

Supplement: Supplementary file 1 — Supplementary Material 1 [file 41065_2025_427_MOESM1_ESM.jpg]
