# Supplementary material for: Mechanistic evaluation of Jiu Wei Qing Zhi Gao in non-alcoholic fatty liver disease: insights from network Pharmacology and experimental validation
Source: Hereditas. 2025 Apr 12;162:59. doi: 10.1186/s41065-025-00427-2 (PMC11992867; doi:10.1186/s41065-025-00427-2)
Supplement: Supplementary file 2 — Supplementary Material 2 [file 41065_2025_427_MOESM2_ESM.docx]

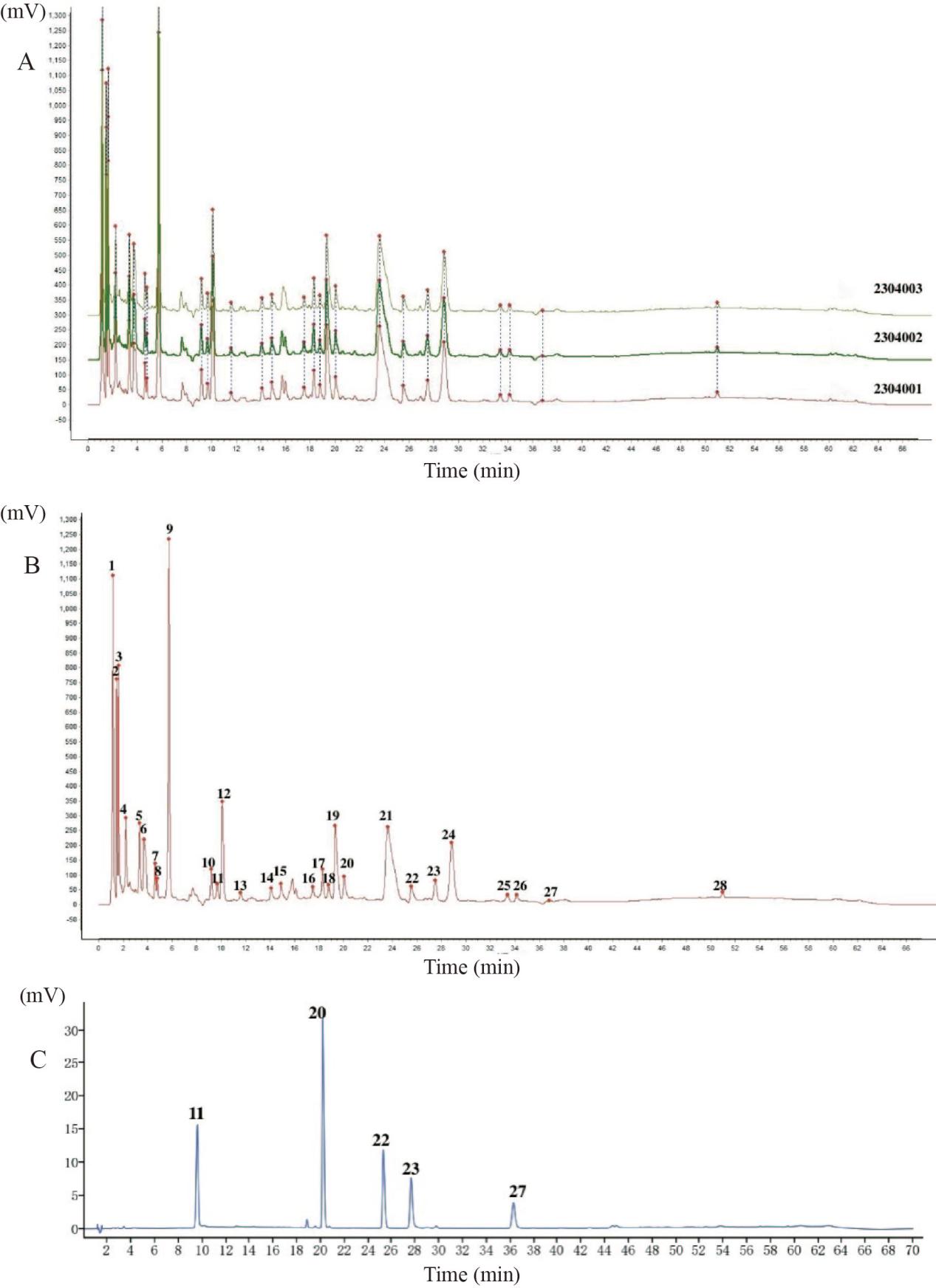


**Supplementary Figure 1.** **Ultra-performance liquid chromatography (UPLC) fingerprint of JWQZG.** (A) The reproducible UPLC chromatograms of JWQZG from 3 batches. (B-C) UPLC chemical fingerprint of JWQZG with 5 peaks determined by comparing retention time with the standards. 11, Paeoniflorin; 20, Epiberberine; 22, Berberine; 23, Quercetin; 27, Kaempferol. JWQZG, Jiu Wei Qing Zhi Gao.

**Supplementary Table 1.** **The bioactive compounds of JWQZG**

| **Ingredient ID** | **Name** | **OB (100%)** | **CAS** | **Network ID** | **Herbal Medicine** |
| --- | --- | --- | --- | --- | --- |
| HBIN018278 | Beta-sitosterol | 36.91 | 83-46-5 | A2 | Sedum sarmentosum Bunge |
| HBIN023519 | DFV | 32.76 | 578-86-9 | CPC2 | Sedum sarmentosum Bunge |
| HBIN033383 | Liquiritin | 65.69 | 551-15-5 | CPC3 | Sedum sarmentosum Bunge |
| HBIN033803 | Luteolin | 36.16 | [491-70-3](https://sso.cas.org/as/authorization.oauth2?response_type=code&client_id=SciFinderWeb&redirect_uri=https://scifinder.cas.org:443/pa/oidc/cb&state=eyJ6aXAiOiJERUYiLCJhbGciOiJkaXIiLCJlbmMiOiJBMTI4Q0JDLUhTMjU2Iiwia2lkIjoiOXkiLCJzdWZmaXgiOiJhMUNGRHEuMTYwMTMwMzQ2MSJ9..X8yiubUlXgmeUjgJJWOgVQ.YnsZwT5nEmHRCz5HU7YSwwsyDOkRvM3B5cZ8lOCqJufEzOFcCRF8IiZM-U0AYdWkW74o3vbMOzef2xqW4-zqNQ.ctWMjsgmXL7mB0JU4AZ08w&nonce=F3WWZ0E1ZhfYL_iNXenE9k48rhSwfVhLoUeZnmopGzo&scope=openid address email phone profile&vnd_pi_requested_resource=https://scifinder.cas.org:443/scifinder&vnd_pi_application_name=SciFinderWebIDF) | CPC4 | Sedum sarmentosum Bunge |
| HBIN034396 | Mangiferonic acid | 40.45 | [13878-90-5](https://sso.cas.org/as/authorization.oauth2?response_type=code&client_id=SciFinderWeb&redirect_uri=https://scifinder.cas.org:443/pa/oidc/cb&state=eyJ6aXAiOiJERUYiLCJhbGciOiJkaXIiLCJlbmMiOiJBMTI4Q0JDLUhTMjU2Iiwia2lkIjoiOXkiLCJzdWZmaXgiOiJhMUNGRHEuMTYwMTMwMzQ2MSJ9..X8yiubUlXgmeUjgJJWOgVQ.YnsZwT5nEmHRCz5HU7YSwwsyDOkRvM3B5cZ8lOCqJufEzOFcCRF8IiZM-U0AYdWkW74o3vbMOzef2xqW4-zqNQ.ctWMjsgmXL7mB0JU4AZ08w&nonce=F3WWZ0E1ZhfYL_iNXenE9k48rhSwfVhLoUeZnmopGzo&scope=openid address email phone profile&vnd_pi_requested_resource=https://scifinder.cas.org:443/scifinder&vnd_pi_application_name=SciFinderWebIDF) | CPC5 | Sedum sarmentosum Bunge |
| HBIN043179 | Sarmentosine_qt | 84.71 | [71933-54-5](https://sso.cas.org/as/authorization.oauth2?response_type=code&client_id=SciFinderWeb&redirect_uri=https://scifinder.cas.org:443/pa/oidc/cb&state=eyJ6aXAiOiJERUYiLCJhbGciOiJkaXIiLCJlbmMiOiJBMTI4Q0JDLUhTMjU2Iiwia2lkIjoiOXkiLCJzdWZmaXgiOiJhMUNGRHEuMTYwMTMwMzQ2MSJ9..X8yiubUlXgmeUjgJJWOgVQ.YnsZwT5nEmHRCz5HU7YSwwsyDOkRvM3B5cZ8lOCqJufEzOFcCRF8IiZM-U0AYdWkW74o3vbMOzef2xqW4-zqNQ.ctWMjsgmXL7mB0JU4AZ08w&nonce=F3WWZ0E1ZhfYL_iNXenE9k48rhSwfVhLoUeZnmopGzo&scope=openid address email phone profile&vnd_pi_requested_resource=https://scifinder.cas.org:443/scifinder&vnd_pi_application_name=SciFinderWebIDF) | CPC6 | Sedum sarmentosum Bunge |
| neosedumoside Ⅱ | Neosedumoside Ⅱ | 49.60 | [480-19-3](https://sso.cas.org/as/authorization.oauth2?response_type=code&client_id=SciFinderWeb&redirect_uri=https://scifinder.cas.org:443/pa/oidc/cb&state=eyJ6aXAiOiJERUYiLCJhbGciOiJkaXIiLCJlbmMiOiJBMTI4Q0JDLUhTMjU2Iiwia2lkIjoiOXkiLCJzdWZmaXgiOiJhMUNGRHEuMTYwMTMwMzQ2MSJ9..X8yiubUlXgmeUjgJJWOgVQ.YnsZwT5nEmHRCz5HU7YSwwsyDOkRvM3B5cZ8lOCqJufEzOFcCRF8IiZM-U0AYdWkW74o3vbMOzef2xqW4-zqNQ.ctWMjsgmXL7mB0JU4AZ08w&nonce=F3WWZ0E1ZhfYL_iNXenE9k48rhSwfVhLoUeZnmopGzo&scope=openid address email phone profile&vnd_pi_requested_resource=https://scifinder.cas.org:443/scifinder&vnd_pi_application_name=SciFinderWebIDF) | CPC7 | Sedum sarmentosum Bunge |
| HBIN031114 | Isorhamnetin | 49.60 | 480-19-3 | CPC8 | Sedum sarmentosum Bunge |
| HBIN041495 | Quercetin | 46.43 | 117-39-5 | A1 | Sedum sarmentosum Bunge |
| HBIN034395 | Mangiferolic acid | 36.16 | [4184-34-3](https://sso.cas.org/as/authorization.oauth2?response_type=code&client_id=SciFinderWeb&redirect_uri=https://scifinder.cas.org:443/pa/oidc/cb&state=eyJ6aXAiOiJERUYiLCJhbGciOiJkaXIiLCJlbmMiOiJBMTI4Q0JDLUhTMjU2Iiwia2lkIjoiOXkiLCJzdWZmaXgiOiJhMUNGRHEuMTYwMTMwMzQ2MSJ9..X8yiubUlXgmeUjgJJWOgVQ.YnsZwT5nEmHRCz5HU7YSwwsyDOkRvM3B5cZ8lOCqJufEzOFcCRF8IiZM-U0AYdWkW74o3vbMOzef2xqW4-zqNQ.ctWMjsgmXL7mB0JU4AZ08w&nonce=F3WWZ0E1ZhfYL_iNXenE9k48rhSwfVhLoUeZnmopGzo&scope=openid address email phone profile&vnd_pi_requested_resource=https://scifinder.cas.org:443/scifinder&vnd_pi_application_name=SciFinderWebIDF) | CPC10 | Sedum sarmentosum Bunge |
| HBIN006367 | 5-Isopropyl-hex-5-enoic acid | 31.07 | [6754-16-1](https://sso.cas.org/as/authorization.oauth2?response_type=code&client_id=SciFinderWeb&redirect_uri=https://scifinder.cas.org:443/pa/oidc/cb&state=eyJ6aXAiOiJERUYiLCJhbGciOiJkaXIiLCJlbmMiOiJBMTI4Q0JDLUhTMjU2Iiwia2lkIjoiOXkiLCJzdWZmaXgiOiJhMUNGRHEuMTYwMTMwMzQ2MSJ9..X8yiubUlXgmeUjgJJWOgVQ.YnsZwT5nEmHRCz5HU7YSwwsyDOkRvM3B5cZ8lOCqJufEzOFcCRF8IiZM-U0AYdWkW74o3vbMOzef2xqW4-zqNQ.ctWMjsgmXL7mB0JU4AZ08w&nonce=F3WWZ0E1ZhfYL_iNXenE9k48rhSwfVhLoUeZnmopGzo&scope=openid address email phone profile&vnd_pi_requested_resource=https://scifinder.cas.org:443/scifinder&vnd_pi_application_name=SciFinderWebIDF) | FL1 | Poria cocos (Schw.) Wolf |
| HBIN006368 | 16alpha-Hydroxydehydrotra-  metenolic acid | 30.93 | [176390-66-2](https://www.chemsrc.com/baike/1468874.html) | FL2 | Poria cocos (Schw.) Wolf |
| HBIN006370 | Polyporenic acid C | 38.26 | [465-18-9](https://sso.cas.org/as/authorization.oauth2?response_type=code&client_id=SciFinderWeb&redirect_uri=https://scifinder.cas.org:443/pa/oidc/cb&state=eyJ6aXAiOiJERUYiLCJhbGciOiJkaXIiLCJlbmMiOiJBMTI4Q0JDLUhTMjU2Iiwia2lkIjoiOXkiLCJzdWZmaXgiOiJhMUNGRHEuMTYwMTMwMzQ2MSJ9..X8yiubUlXgmeUjgJJWOgVQ.YnsZwT5nEmHRCz5HU7YSwwsyDOkRvM3B5cZ8lOCqJufEzOFcCRF8IiZM-U0AYdWkW74o3vbMOzef2xqW4-zqNQ.ctWMjsgmXL7mB0JU4AZ08w&nonce=F3WWZ0E1ZhfYL_iNXenE9k48rhSwfVhLoUeZnmopGzo&scope=openid address email phone profile&vnd_pi_requested_resource=https://scifinder.cas.org:443/scifinder&vnd_pi_application_name=SciFinderWebIDF) | FL3 | Poria cocos (Schw.) Wolf |
| HBIN008225 | 3beta-Hydroxy-24-methylene-8-  lanostene-21-oic acid | 38.70 | 560-66-7 | FL4 | Poria cocos (Schw.) Wolf |
| HBIN012997 | 7,9(11)-Dehydropachymic acid | 35.11 | [77012-31-8](https://sso.cas.org/as/authorization.oauth2?response_type=code&client_id=SciFinderWeb&redirect_uri=https://scifinder.cas.org:443/pa/oidc/cb&state=eyJ6aXAiOiJERUYiLCJhbGciOiJkaXIiLCJlbmMiOiJBMTI4Q0JDLUhTMjU2Iiwia2lkIjoiOXkiLCJzdWZmaXgiOiJhMUNGRHEuMTYwMTMwMzQ2MSJ9..X8yiubUlXgmeUjgJJWOgVQ.YnsZwT5nEmHRCz5HU7YSwwsyDOkRvM3B5cZ8lOCqJufEzOFcCRF8IiZM-U0AYdWkW74o3vbMOzef2xqW4-zqNQ.ctWMjsgmXL7mB0JU4AZ08w&nonce=F3WWZ0E1ZhfYL_iNXenE9k48rhSwfVhLoUeZnmopGzo&scope=openid address email phone profile&vnd_pi_requested_resource=https://scifinder.cas.org:443/scifinder&vnd_pi_application_name=SciFinderWebIDF) | FL5 | Poria cocos (Schw.) Wolf |
| HBIN013153 | 7-Dehydrosigmasterol | 37.42 | 6869-99-4 | FL6 | Poria cocos (Schw.) Wolf |
| HBIN020146 | Cerevisterol | 37.96 | [516-37-0](https://sso.cas.org/as/authorization.oauth2?response_type=code&client_id=SciFinderWeb&redirect_uri=https://scifinder.cas.org:443/pa/oidc/cb&state=eyJ6aXAiOiJERUYiLCJhbGciOiJkaXIiLCJlbmMiOiJBMTI4Q0JDLUhTMjU2Iiwia2lkIjoiOXkiLCJzdWZmaXgiOiJhMUNGRHEuMTYwMTMwMzQ2MSJ9..X8yiubUlXgmeUjgJJWOgVQ.YnsZwT5nEmHRCz5HU7YSwwsyDOkRvM3B5cZ8lOCqJufEzOFcCRF8IiZM-U0AYdWkW74o3vbMOzef2xqW4-zqNQ.ctWMjsgmXL7mB0JU4AZ08w&nonce=F3WWZ0E1ZhfYL_iNXenE9k48rhSwfVhLoUeZnmopGzo&scope=openid address email phone profile&vnd_pi_requested_resource=https://scifinder.cas.org:443/scifinder&vnd_pi_application_name=SciFinderWebIDF) | FL7 | Poria cocos (Schw.) Wolf |
| HBIN020260 | Cheilanthifoline | 46.51 | [483-44-3](https://sso.cas.org/as/authorization.oauth2?response_type=code&client_id=SciFinderWeb&redirect_uri=https://scifinder.cas.org:443/pa/oidc/cb&state=eyJ6aXAiOiJERUYiLCJhbGciOiJkaXIiLCJlbmMiOiJBMTI4Q0JDLUhTMjU2Iiwia2lkIjoiOXkiLCJzdWZmaXgiOiJhMUNGRHEuMTYwMTMwMzQ2MSJ9..X8yiubUlXgmeUjgJJWOgVQ.YnsZwT5nEmHRCz5HU7YSwwsyDOkRvM3B5cZ8lOCqJufEzOFcCRF8IiZM-U0AYdWkW74o3vbMOzef2xqW4-zqNQ.ctWMjsgmXL7mB0JU4AZ08w&nonce=F3WWZ0E1ZhfYL_iNXenE9k48rhSwfVhLoUeZnmopGzo&scope=openid address email phone profile&vnd_pi_requested_resource=https://scifinder.cas.org:443/scifinder&vnd_pi_application_name=SciFinderWebIDF) | FL8 | Poria cocos (Schw.) Wolf |
| HBIN023022 | Dehydroeburicoic acid | 44.17 | 1818670 | FL9 | Poria cocos (Schw.) Wolf |
| HBIN025006 | Ellagic acid | 43.06 | [476-66-4](https://sso.cas.org/as/authorization.oauth2?response_type=code&client_id=SciFinderWeb&redirect_uri=https://scifinder.cas.org:443/pa/oidc/cb&state=eyJ6aXAiOiJERUYiLCJhbGciOiJkaXIiLCJlbmMiOiJBMTI4Q0JDLUhTMjU2Iiwia2lkIjoiOXkiLCJzdWZmaXgiOiJhMUNGRHEuMTYwMTMwMzQ2MSJ9..X8yiubUlXgmeUjgJJWOgVQ.YnsZwT5nEmHRCz5HU7YSwwsyDOkRvM3B5cZ8lOCqJufEzOFcCRF8IiZM-U0AYdWkW74o3vbMOzef2xqW4-zqNQ.ctWMjsgmXL7mB0JU4AZ08w&nonce=F3WWZ0E1ZhfYL_iNXenE9k48rhSwfVhLoUeZnmopGzo&scope=openid address email phone profile&vnd_pi_requested_resource=https://scifinder.cas.org:443/scifinder&vnd_pi_application_name=SciFinderWebIDF) | FL10 | Poria cocos (Schw.) Wolf |
| HBIN025012 | Ellipticine | 30.82 | 519-23-3 | FL11 | Poria cocos (Schw.) Wolf |
| HBIN025543 | Ergosta-7,22E-dien-3beta-ol | 43.51 | [1105-11-9](https://sso.cas.org/as/authorization.oauth2?response_type=code&client_id=SciFinderWeb&redirect_uri=https://scifinder.cas.org:443/pa/oidc/cb&state=eyJ6aXAiOiJERUYiLCJhbGciOiJkaXIiLCJlbmMiOiJBMTI4Q0JDLUhTMjU2Iiwia2lkIjoiOXkiLCJzdWZmaXgiOiJhMUNGRHEuMTYwMTMwMzQ2MSJ9..X8yiubUlXgmeUjgJJWOgVQ.YnsZwT5nEmHRCz5HU7YSwwsyDOkRvM3B5cZ8lOCqJufEzOFcCRF8IiZM-U0AYdWkW74o3vbMOzef2xqW4-zqNQ.ctWMjsgmXL7mB0JU4AZ08w&nonce=F3WWZ0E1ZhfYL_iNXenE9k48rhSwfVhLoUeZnmopGzo&scope=openid address email phone profile&vnd_pi_requested_resource=https://scifinder.cas.org:443/scifinder&vnd_pi_application_name=SciFinderWebIDF) | FL12 | Poria cocos (Schw.) Wolf |
| **Supplementary Table 1. Continued** | | | | | |
| **Ingredient ID** | **Name** | **OB (100%)** | **CAS** | **Network ID** | **Herbal Medicine** |
| HBIN025557 | Ergosterol peroxide | 40.36 | 138329-20-1 | FL13 | Poria cocos (Schw.) Wolf |
| HBIN027035 | Gallicacid-3-O-(6'-O-galloyl)-glucoside | 30.25 | 87087-61-4 | B1 | Poria cocos (Schw.) Wolf |
| HBIN028847 | Hederagenin | 36.91 | 465-99-6 | FL15 | Poria cocos (Schw.) Wolf |
| HBIN038556 | Pachymic acid | 33.63 | [29070-92-6](https://sso.cas.org/as/authorization.oauth2?response_type=code&client_id=SciFinderWeb&redirect_uri=https://scifinder.cas.org:443/pa/oidc/cb&state=eyJ6aXAiOiJERUYiLCJhbGciOiJkaXIiLCJlbmMiOiJBMTI4Q0JDLUhTMjU2Iiwia2lkIjoiOXkiLCJzdWZmaXgiOiJhMUNGRHEuMTYwMTMwMzQ2MSJ9..X8yiubUlXgmeUjgJJWOgVQ.YnsZwT5nEmHRCz5HU7YSwwsyDOkRvM3B5cZ8lOCqJufEzOFcCRF8IiZM-U0AYdWkW74o3vbMOzef2xqW4-zqNQ.ctWMjsgmXL7mB0JU4AZ08w&nonce=F3WWZ0E1ZhfYL_iNXenE9k48rhSwfVhLoUeZnmopGzo&scope=openid address email phone profile&vnd_pi_requested_resource=https://scifinder.cas.org:443/scifinder&vnd_pi_application_name=SciFinderWebIDF) | FL16 | Poria cocos (Schw.) Wolf |
| HBIN038558 | Pachypodol | 75.06 | 33708-72-4 | FL17 | Poria cocos (Schw.) Wolf |
| HBIN039207 | Peraksine | 82.58 | [15527-80-7](https://sso.cas.org/as/authorization.oauth2?response_type=code&client_id=SciFinderWeb&redirect_uri=https://scifinder.cas.org:443/pa/oidc/cb&state=eyJ6aXAiOiJERUYiLCJhbGciOiJkaXIiLCJlbmMiOiJBMTI4Q0JDLUhTMjU2Iiwia2lkIjoiOXkiLCJzdWZmaXgiOiJhMUNGRHEuMTYwMTMwMzQ2MSJ9..X8yiubUlXgmeUjgJJWOgVQ.YnsZwT5nEmHRCz5HU7YSwwsyDOkRvM3B5cZ8lOCqJufEzOFcCRF8IiZM-U0AYdWkW74o3vbMOzef2xqW4-zqNQ.ctWMjsgmXL7mB0JU4AZ08w&nonce=F3WWZ0E1ZhfYL_iNXenE9k48rhSwfVhLoUeZnmopGzo&scope=openid address email phone profile&vnd_pi_requested_resource=https://scifinder.cas.org:443/scifinder&vnd_pi_application_name=SciFinderWebIDF) | FL18 | Poria cocos (Schw.) Wolf |
| HBIN040570 | Poricoic acid A | 30.61 | [137551-38-3](https://sso.cas.org/as/authorization.oauth2?response_type=code&client_id=SciFinderWeb&redirect_uri=https://scifinder.cas.org:443/pa/oidc/cb&state=eyJ6aXAiOiJERUYiLCJhbGciOiJkaXIiLCJlbmMiOiJBMTI4Q0JDLUhTMjU2Iiwia2lkIjoiOXkiLCJzdWZmaXgiOiJhMUNGRHEuMTYwMTMwMzQ2MSJ9..X8yiubUlXgmeUjgJJWOgVQ.YnsZwT5nEmHRCz5HU7YSwwsyDOkRvM3B5cZ8lOCqJufEzOFcCRF8IiZM-U0AYdWkW74o3vbMOzef2xqW4-zqNQ.ctWMjsgmXL7mB0JU4AZ08w&nonce=F3WWZ0E1ZhfYL_iNXenE9k48rhSwfVhLoUeZnmopGzo&scope=openid address email phone profile&vnd_pi_requested_resource=https://scifinder.cas.org:443/scifinder&vnd_pi_application_name=SciFinderWebIDF) | FL19 | Poria cocos (Schw.) Wolf |
| HBIN040573 | Poricoic acid B | 30.52 | [137551-39-4](https://sso.cas.org/as/authorization.oauth2?response_type=code&client_id=SciFinderWeb&redirect_uri=https://scifinder.cas.org:443/pa/oidc/cb&state=eyJ6aXAiOiJERUYiLCJhbGciOiJkaXIiLCJlbmMiOiJBMTI4Q0JDLUhTMjU2Iiwia2lkIjoiOXkiLCJzdWZmaXgiOiJhMUNGRHEuMTYwMTMwMzQ2MSJ9..X8yiubUlXgmeUjgJJWOgVQ.YnsZwT5nEmHRCz5HU7YSwwsyDOkRvM3B5cZ8lOCqJufEzOFcCRF8IiZM-U0AYdWkW74o3vbMOzef2xqW4-zqNQ.ctWMjsgmXL7mB0JU4AZ08w&nonce=F3WWZ0E1ZhfYL_iNXenE9k48rhSwfVhLoUeZnmopGzo&scope=openid address email phone profile&vnd_pi_requested_resource=https://scifinder.cas.org:443/scifinder&vnd_pi_application_name=SciFinderWebIDF) | FL20 | Poria cocos (Schw.) Wolf |
| HBIN040575 | poricoic acid C | 38.15 | [151200-89-4](https://sso.cas.org/as/authorization.oauth2?response_type=code&client_id=SciFinderWeb&redirect_uri=https://scifinder.cas.org:443/pa/oidc/cb&state=eyJ6aXAiOiJERUYiLCJhbGciOiJkaXIiLCJlbmMiOiJBMTI4Q0JDLUhTMjU2Iiwia2lkIjoiOXkiLCJzdWZmaXgiOiJhMUNGRHEuMTYwMTMwMzQ2MSJ9..X8yiubUlXgmeUjgJJWOgVQ.YnsZwT5nEmHRCz5HU7YSwwsyDOkRvM3B5cZ8lOCqJufEzOFcCRF8IiZM-U0AYdWkW74o3vbMOzef2xqW4-zqNQ.ctWMjsgmXL7mB0JU4AZ08w&nonce=F3WWZ0E1ZhfYL_iNXenE9k48rhSwfVhLoUeZnmopGzo&scope=openid address email phone profile&vnd_pi_requested_resource=https://scifinder.cas.org:443/scifinder&vnd_pi_application_name=SciFinderWebIDF) | FL21 | Poria cocos (Schw.) Wolf |
| HBIN046625 | Trametenolic acid | 38.71 | [24160-36-9](https://sso.cas.org/as/authorization.oauth2?response_type=code&client_id=SciFinderWeb&redirect_uri=https://scifinder.cas.org:443/pa/oidc/cb&state=eyJ6aXAiOiJERUYiLCJhbGciOiJkaXIiLCJlbmMiOiJBMTI4Q0JDLUhTMjU2Iiwia2lkIjoiOXkiLCJzdWZmaXgiOiJhMUNGRHEuMTYwMTMwMzQ2MSJ9..X8yiubUlXgmeUjgJJWOgVQ.YnsZwT5nEmHRCz5HU7YSwwsyDOkRvM3B5cZ8lOCqJufEzOFcCRF8IiZM-U0AYdWkW74o3vbMOzef2xqW4-zqNQ.ctWMjsgmXL7mB0JU4AZ08w&nonce=F3WWZ0E1ZhfYL_iNXenE9k48rhSwfVhLoUeZnmopGzo&scope=openid address email phone profile&vnd_pi_requested_resource=https://scifinder.cas.org:443/scifinder&vnd_pi_application_name=SciFinderWebIDF) | FL22 | Poria cocos (Schw.) Wolf |
| HBIN041752 | Quinicine | 75.44 | 52211-63-9 | FL23 | Poria cocos (Schw.) Wolf |
| HBIN017893 | Berberine | 36.86 | 2086-83-1 | HL1 | Coptis chinensis Franch |
| HBIN017897 | Berberrubine | 35.74 | 15401-69-1 | HL2 | Coptis chinensis Franch |
| HBIN017927 | Berlambine | 36.68 | 549-21-3 | HL3 | Coptis chinensis Franch |
| HBIN021430 | Coptisine | 30.67 | 3486-66-6 | HL4 | Coptis chinensis Franch |
| HBIN021443 | Corchoroside A_qt | 104.95 | NA | HL5 | Coptis chinensis Franch |
| HBIN024823 | Eckol | 87.06 | 88798-74-7 | HL6 | Coptis chinensis Franch |
| HBIN025268 | Epiberberine | 43.09 | 1816598 | HL7 | Coptis chinensis Franch |
| HBIN031499 | Jatrorrhizine | 30.44 | 3621-38-3 | HL8 | Coptis chinensis Franch |
| HBIN034220 | Magnograndiolide | 63.71 | 92618-98-9 | HL9 | Coptis chinensis Franch |
| HBIN035808 | Moupinamide | 86.71 | 66648-43-9 | HL10 | Coptis chinensis Franch |
| HBIN037642 | Obacunone | 81.58 | 751-03-1 | HL11 | Coptis chinensis Franch |
| HBIN038666 | Palmatine | 64.60 | 3486-67-7 | HL12 | Coptis chinensis Franch |
| HBIN038674 | Palmidin A | 35.36 | NA | HL13 | Coptis chinensis Franch |
| **Supplementary Table 1. Continued** | | | | | |
| **Ingredient ID** | **Name** | **OB (100%)** | **CAS** | **Network ID** | **Herbal Medicine** |
| HBIN041495 | Quercetin | 46.43 | 117-39-5 | A1 | Coptis chinensis Franch |
| HBIN041970 | (R)-Canadine | 55.37 | 522-97-4 | HL15 | Coptis chinensis Franch |
| Worenine | Worenine | 52.94 | [1678-97-3](https://sso.cas.org/as/authorization.oauth2?response_type=code&client_id=SciFinderWeb&redirect_uri=https://scifinder.cas.org:443/pa/oidc/cb&state=eyJ6aXAiOiJERUYiLCJhbGciOiJkaXIiLCJlbmMiOiJBMTI4Q0JDLUhTMjU2Iiwia2lkIjoiOXkiLCJzdWZmaXgiOiJhMUNGRHEuMTYwMTMwMzQ2MSJ9..X8yiubUlXgmeUjgJJWOgVQ.YnsZwT5nEmHRCz5HU7YSwwsyDOkRvM3B5cZ8lOCqJufEzOFcCRF8IiZM-U0AYdWkW74o3vbMOzef2xqW4-zqNQ.ctWMjsgmXL7mB0JU4AZ08w&nonce=F3WWZ0E1ZhfYL_iNXenE9k48rhSwfVhLoUeZnmopGzo&scope=openid address email phone profile&vnd_pi_requested_resource=https://scifinder.cas.org:443/scifinder&vnd_pi_application_name=SciFinderWebIDF) | HL16 | Coptis chinensis Franch |
| HBIN009663 | Palbinone | 43.56 | 139954-00-0 | BS1 | Paeonia lactiflora Pall |
| HBIN015226 | Alloisoimperatorin | 34.80 | 35214-83-6 | BS2 | Paeonia lactiflora Pall |
| HBIN017827 | Benzoyl paeoniflorin | 31.27 | 38642-49-8 | BS4 | Paeonia lactiflora Pall |
| HBIN018278 | Beta-sitosterol | 36.91 | 83-46-5 | A2 | Paeonia lactiflora Pall |
| HBIN019919 | (-)-catechin | 49.68 | 154-23-4 | BS5 | Paeonia lactiflora Pall |
| HBIN019921 | (+)-catechin | 54.83 | 154-23-4 | BS6 | Paeonia lactiflora Pall |
| HBIN030819 | Isoimperatorin | 45.46 | 482-45-1 | BS7 | Paeonia lactiflora Pall |
| HBIN031753 | Kaempferol | 41.88 | 520-18-3 | BS8 | Paeonia lactiflora Pall |
| HBIN034281 | Mairin | 55.38 | 472-15-1 | BS9 | Paeonia lactiflora Pall |
| HBIN034507 | Marmesine | 84.77 | 495-32-9 | BS10 | Paeonia lactiflora Pall |
| HBIN035198 | Methylenetanshinquinone | 37.07 | 67656-29-5 | BS11 | Paeonia lactiflora Pall |
| HBIN036568 | Neobyakangelico l | 36.18 | 35214-82-5 | BS12 | Paeonia lactiflora Pall |
| HBIN037195 | Nodakenin | 57.12 | 495-31-8 | BS13 | Paeonia lactiflora Pall |
| HBIN038604 | Paeoniflorgenone | 87.59 | 80454-42-8 | BS14 | Paeonia lactiflora Pall |
| HBIN038606 | Paeoniflorin | 53.87 | 23180-57-6 | BS15 | Paeonia lactiflora Pall |
| HBIN039451 | Phellopterin | 40.19 | 2543-94-4 | BS16 | Paeonia lactiflora Pall |
| HBIN041379 | Pyrethrin II | 48.36 | 121-29-9 | BS17 | Paeonia lactiflora Pall |
| HBIN043688 | Sen-byakangelicol | 58.00 | NA | BS18 | Paeonia lactiflora Pall |
| HBIN018006 | Beta carotene | 37.18 | 7235-40-7 | CS1 | Faeces Bombycis |
| HBIN018163 | Betaine | 40.92 | 107-43-7 | CS2 | Faeces Bombycis |
| HBIN021150 | CLR | 37.87 | 80356-14-5 | C1 | Faeces Bombycis |
| **Supplementary Table 1. Continued** | | | | | |
| **Ingredient ID** | **Name** | **OB (100%)** | **CAS** | **Network ID** | **Herbal Medicine** |
| HBIN023809 | Dihydroanhydropodorhizol | 48.29 | 40456-50-6 | CS4 | Faeces Bombycis |
| HBIN026332 | FA | 68.96 | 33609-88-0 | CS5 | Faeces Bombycis |
| HBIN000624 | 1,2,3-Trimethylcyclohexane | 52.94 | 1678-97-3 | SZ1 | Crataegus pinnatifida var. pinnatifida |
| HBIN001034 | 1,3,4-Trimethyl-3-cyclohexene-1-  carboxaldehyde | 56.58 | 40702-26-9 | SZ2 | Crataegus pinnatifida var. pinnatifida |
| HBIN003899 | 2,3,4-Trimethylhexane | 38.91 | [921-47-1](https://sso.cas.org/as/authorization.oauth2?response_type=code&client_id=SciFinderWeb&redirect_uri=https://scifinder.cas.org:443/pa/oidc/cb&state=eyJ6aXAiOiJERUYiLCJhbGciOiJkaXIiLCJlbmMiOiJBMTI4Q0JDLUhTMjU2Iiwia2lkIjoiOXkiLCJzdWZmaXgiOiJhMUNGRHEuMTYwMTMwMzQ2MSJ9..X8yiubUlXgmeUjgJJWOgVQ.YnsZwT5nEmHRCz5HU7YSwwsyDOkRvM3B5cZ8lOCqJufEzOFcCRF8IiZM-U0AYdWkW74o3vbMOzef2xqW4-zqNQ.ctWMjsgmXL7mB0JU4AZ08w&nonce=F3WWZ0E1ZhfYL_iNXenE9k48rhSwfVhLoUeZnmopGzo&scope=openid address email phone profile&vnd_pi_requested_resource=https://scifinder.cas.org:443/scifinder&vnd_pi_application_name=SciFinderWebIDF) | SZ3 | Crataegus pinnatifida var. pinnatifida |
| HBIN004064 | 2,3-Dimethylheptane | 35.13 | 3074-71-3 | SZ4 | Crataegus pinnatifida var. pinnatifida |
| HBIN008949 | 3-Methylhexane | 38.19 | 589-34-4 | SZ5 | Crataegus pinnatifida var. pinnatifida |
| HBIN020984 | citric acid | 56.22 | [77-92-9](https://sso.cas.org/as/authorization.oauth2?response_type=code&client_id=SciFinderWeb&redirect_uri=https://scifinder.cas.org:443/pa/oidc/cb&state=eyJ6aXAiOiJERUYiLCJhbGciOiJkaXIiLCJlbmMiOiJBMTI4Q0JDLUhTMjU2Iiwia2lkIjoiOXkiLCJzdWZmaXgiOiJhMUNGRHEuMTYwMTMwMzQ2MSJ9..X8yiubUlXgmeUjgJJWOgVQ.YnsZwT5nEmHRCz5HU7YSwwsyDOkRvM3B5cZ8lOCqJufEzOFcCRF8IiZM-U0AYdWkW74o3vbMOzef2xqW4-zqNQ.ctWMjsgmXL7mB0JU4AZ08w&nonce=F3WWZ0E1ZhfYL_iNXenE9k48rhSwfVhLoUeZnmopGzo&scope=openid address email phone profile&vnd_pi_requested_resource=https://scifinder.cas.org:443/scifinder&vnd_pi_application_name=SciFinderWebIDF) | SZ6 | Crataegus pinnatifida var. pinnatifida |
| HBIN025906 | Ethylcyclohexane | 54.13 | 1678-91-7 | SZ7 | Crataegus pinnatifida var. pinnatifida |
| HBIN033339 | Linolenic acid | 45.01 | 463-40-1 | SZ8 | Crataegus pinnatifida var. pinnatifida |
| HBIN033346 | Linoleyl acetate | 42.10 | 5999-95-1 | SZ9 | Crataegus pinnatifida var. pinnatifida |
| HBIN045071 | Suchilactone | 57.52 | 50816-74-5 | SZ10 | Crataegus pinnatifida var. pinnatifida |
| HBIN016113 | Angeloylgomisin O | 31.97 | NA | WWZ1 | Schisandra chinensis (Turcz.) Baill |
| HBIN028275 | Gomisin B | 31.99 | 58546-55-7 | WWZ2 | Schisandra chinensis (Turcz.) Baill |
| HBIN028280 | Gomisin G | 32.68 | NA | WWZ3 | Schisandra chinensis (Turcz.) Baill |
| HBIN033504 | Longikaurin A | 47.72 | 75207-67-9 | WWZ4 | Schisandra chinensis (Turcz.) Baill |
| HBIN048412 | Wuweizisu C | 46.27 | 61301-33-5 | WWZ5 | Schisandra chinensis (Turcz.) Baill |
| HBIN043358 | Schizandrer B | 30.71 | 82078-76-0 | WWZ6 | Schisandra chinensis (Turcz.) Baill |
| HBIN028274 | Gomisin-A | 30.69 | 58546-54-6 | WWZ7 | Schisandra chinensis (Turcz.) Baill |
| HBIN023368 | Deoxyharringtonine | 39.27 | 36804-95-2 | WWZ8 | Schisandra chinensis (Turcz.) Baill |
| HBIN006106 | 2-Monoolein | 34.23 | 3443-84-3 | YR1 | Coix lacryma-jobi var. ma-yuen (Rom.Caill.) Stapf |
| HBIN006366 | [3-Oleoyl-sn-glycerol](https://www.ncbi.nlm.nih.gov/pcsubstance/?term="3-oleoyl-sn-glycerol"[CompleteSynonym] AND 11451146[StandardizedCID]" \t "https://pubchem.ncbi.nlm.nih.gov/compound/_self) | 34.13 | 111-03-5 | YR2 | Coix lacryma-jobi var. ma-yuen (Rom.Caill.) Stapf |
| **Supplementary Table 1. Continued** | | | | | |
| **Ingredient ID** | **Name** | **OB (100%)** | **CAS** | **Network ID** | **Herbal Medicine** |
| HBIN018278 | Beta-sitosterol | 36.91 | 83-46-5 | A2 | Coix lacryma-jobi var. ma-yuen (Rom.Caill.) Stapf |
| HBIN021150 | CLR | 37.87 | 80356-14-5 | C1 | Coix lacryma-jobi var. ma-yuen (Rom.Caill.) Stapf |
| HBIN012837 | [Hydrosqualene](https://www.ncbi.nlm.nih.gov/pcsubstance/?term="hydrosqualene"[CompleteSynonym] AND 11975273[StandardizedCID]" \t "https://pubchem.ncbi.nlm.nih.gov/compound/_self) | 33.55 | 7683-64-9 | YR3 | Coix lacryma-jobi var. ma-yuen (Rom.Caill.) Stapf |
| HBIN034389 | Mandenol | 42.00 | 544-35-4 | YR6 | Coix lacryma-jobi var. ma-yuen (Rom.Caill.) Stapf |
| HBIN044161 | Sitosterol alpha1 | 43.28 | 474-40-8 | YR7 | Coix lacryma-jobi var. ma-yuen (Rom.Caill.) Stapf |
| HBIN044918 | Stigmasterol | 43.82 | 83-48-7 | YR8 | Coix lacryma-jobi var. ma-yuen (Rom.Caill.) Stapf |
| HBIN021254 | Coixenolide | 32.39 | 29066-43-1 | YR9 | Coix lacryma-jobi var. ma-yuen (Rom.Caill.) Stapf |
| HBIN018278 | Beta-sitosterol | 36.91 | 83-46-5 | A2 | Lycopus lucidus Turcz. ex Benth |
| HBIN019296 | Caffeate | 54.97 | 331-39-5 | ZL2 | Lycopus lucidus Turcz. ex Benth |
| HBIN022815 | DBP | 64.54 | [84-74-2](https://sso.cas.org/as/authorization.oauth2?response_type=code&client_id=SciFinderWeb&redirect_uri=https://scifinder.cas.org:443/pa/oidc/cb&state=eyJ6aXAiOiJERUYiLCJhbGciOiJkaXIiLCJlbmMiOiJBMTI4Q0JDLUhTMjU2Iiwia2lkIjoiOXkiLCJzdWZmaXgiOiJhMUNGRHEuMTYwMTMwMzQ2MSJ9..X8yiubUlXgmeUjgJJWOgVQ.YnsZwT5nEmHRCz5HU7YSwwsyDOkRvM3B5cZ8lOCqJufEzOFcCRF8IiZM-U0AYdWkW74o3vbMOzef2xqW4-zqNQ.ctWMjsgmXL7mB0JU4AZ08w&nonce=F3WWZ0E1ZhfYL_iNXenE9k48rhSwfVhLoUeZnmopGzo&scope=openid address email phone profile&vnd_pi_requested_resource=https://scifinder.cas.org:443/scifinder&vnd_pi_application_name=SciFinderWebIDF) | ZL3 | Lycopus lucidus Turcz. ex Benth |
| HBIN028847 | Hederagenin | 36.91 | [465-99-6](https://sso.cas.org/as/authorization.oauth2?response_type=code&client_id=SciFinderWeb&redirect_uri=https://scifinder.cas.org:443/pa/oidc/cb&state=eyJ6aXAiOiJERUYiLCJhbGciOiJkaXIiLCJlbmMiOiJBMTI4Q0JDLUhTMjU2Iiwia2lkIjoiOXkiLCJzdWZmaXgiOiJhMUNGRHEuMTYwMTMwMzQ2MSJ9..X8yiubUlXgmeUjgJJWOgVQ.YnsZwT5nEmHRCz5HU7YSwwsyDOkRvM3B5cZ8lOCqJufEzOFcCRF8IiZM-U0AYdWkW74o3vbMOzef2xqW4-zqNQ.ctWMjsgmXL7mB0JU4AZ08w&nonce=F3WWZ0E1ZhfYL_iNXenE9k48rhSwfVhLoUeZnmopGzo&scope=openid address email phone profile&vnd_pi_requested_resource=https://scifinder.cas.org:443/scifinder&vnd_pi_application_name=SciFinderWebIDF) | B1 | Lycopus lucidus Turcz. ex Benth |
| HBIN040905 | Protocatechualdehyde | 38.35 | [139-85-5](https://sso.cas.org/as/authorization.oauth2?response_type=code&client_id=SciFinderWeb&redirect_uri=https://scifinder.cas.org:443/pa/oidc/cb&state=eyJ6aXAiOiJERUYiLCJhbGciOiJkaXIiLCJlbmMiOiJBMTI4Q0JDLUhTMjU2Iiwia2lkIjoiOXkiLCJzdWZmaXgiOiJhMUNGRHEuMTYwMTMwMzQ2MSJ9..X8yiubUlXgmeUjgJJWOgVQ.YnsZwT5nEmHRCz5HU7YSwwsyDOkRvM3B5cZ8lOCqJufEzOFcCRF8IiZM-U0AYdWkW74o3vbMOzef2xqW4-zqNQ.ctWMjsgmXL7mB0JU4AZ08w&nonce=F3WWZ0E1ZhfYL_iNXenE9k48rhSwfVhLoUeZnmopGzo&scope=openid address email phone profile&vnd_pi_requested_resource=https://scifinder.cas.org:443/scifinder&vnd_pi_application_name=SciFinderWebIDF) | ZL5 | Lycopus lucidus Turcz. ex Benth |
| HBIN046584 | Torulene | 33.49 | [547-23-9](https://sso.cas.org/as/authorization.oauth2?response_type=code&client_id=SciFinderWeb&redirect_uri=https://scifinder.cas.org:443/pa/oidc/cb&state=eyJ6aXAiOiJERUYiLCJhbGciOiJkaXIiLCJlbmMiOiJBMTI4Q0JDLUhTMjU2Iiwia2lkIjoiOXkiLCJzdWZmaXgiOiJhMUNGRHEuMTYwMTMwMzQ2MSJ9..X8yiubUlXgmeUjgJJWOgVQ.YnsZwT5nEmHRCz5HU7YSwwsyDOkRvM3B5cZ8lOCqJufEzOFcCRF8IiZM-U0AYdWkW74o3vbMOzef2xqW4-zqNQ.ctWMjsgmXL7mB0JU4AZ08w&nonce=F3WWZ0E1ZhfYL_iNXenE9k48rhSwfVhLoUeZnmopGzo&scope=openid address email phone profile&vnd_pi_requested_resource=https://scifinder.cas.org:443/scifinder&vnd_pi_application_name=SciFinderWebIDF) | ZL6 | Lycopus lucidus Turcz. ex Benth |

**Supplementary Table 2. The Targets of Jiu Wei Qing Zhi Gao**

| Name | Name | Name | Name |
| --- | --- | --- | --- |
| ABCB1 | SIRT1 | CHEK1 | CAMK2B |
| ABCC1 | SIRT3 | CDK8 | GPR35 |
| ABCC8 | SLC10A1 | PDE1A | MAPT |
| ABCG2 | SLC10A2 | PDE1B | APEX1 |
| ACACA | SLC18A2 | PDE1C | PTPRS |
| ACACB | SLC22A6 | CSF1R | MPG |
| ACE | SLC5A2 | NOS1 | CDK6 |
| ADH1A | SLC6A3 | QPCTL | HTR1A |
| ADH1B | SLC6A4 | BAD | DRD5 |
| ADH1C | SNCA | CCNA1 | KCNN3 |
| ADH4 | SRC | TYK2 | CHRNB4 |
| ADORA2A | SREBF2 | ROCK2 | CHRNA2 |
| ADRA2A | ST6GAL1 | ROCK1 | CHRNB2 |
| ADRB1 | STAT3 | MKNK1 | CHRNA3 |
| ADRB2 | STS | HTR2C | CHRNA4 |
| ADRB3 | SYK | ACVRL1 | KCNN1 |
| AGTR1 | TACR1 | PLK1 | KCNN2 |
| AHR | TERT | PLK2 | HTR1B |
| AKR1A1 | TGFB1 | STK17B | NEK1 |
| AKR1B1 | TGFBR1 | STK17A | TDP2 |
| AKR1B10 | TGFBR2 | MAPKAPK2 | SAE1 |
| AKT1 | TGM2 | HDAC6 | ADK |
| ALDH1A1 | THRB | P2RX3 | PTGDR |
| ALDH2 | TLR4 | NTRK1 | ITGA4 |
| ALDH3A1 | TLR9 | ROS1 | PTGER3 |
| ALK | TNF | BRPF1 | RASGRP3 |
| ALOX5 | TNFRSF1A | ABCC9 | S1PR2 |
| AOC3 | TTR | KLK5 | CYSLTR1 |
| APP | VCP | PIM3 | HAO1 |
| AR | VEGFA | GPR139 | LNPEP |
| ARG1 | XBP1 | LRRK2 | PLCG1 |
| ATM | XDH | OPRL1 | PTGFR |
| AURKA | XIAP | OPRM1 | ITGB3 |
| AURKB | SLC6A2 | OPRK1 | ITGAV |
| BCL2 | BCHE | CDK7 | PLEC |
| BCL2L1 | SERPINA6 | CCNH | NTSR1 |
| BIRC2 | CHRM2 | CDK9 | ITGB7 |
| BRAF | ACHE | CCNT1 | P2RY12 |
| CACNA2D1 | VDR | NCOR2 | EDNRB |
| CASP3 | PTGER1 | CCKAR | UBLCP1 |
| CYP51A1 | PTGER2 | FLT3 | SGK1 |
| CASP7 | DHCR7 | HDAC4 | ITGA2B |
| CASP8 | GLRA1 | TK1 | FKBP5 |
| CASP9 | SQLE | MKNK2 | FKBP4 |
| CAT | PTPN6 | TUBB1 | HSF1 |
| CCNA2 | NR3C1 | F9 | ECE1 |
| CCNB1 | CDC25B | RET | STAT5B |
| CCND1 | UGT2B7 | CCNB3 | CASR |
| CCNE1 | HSD11B2 | CCNB2 | GC |
| CCR1 | POLB | GPR88 | ACKR3 |
| CCR5 | CA2 | CX3CR1 | MAPK13 |
| CCR9 | CA7 | CAPN1 | GRIN2A |
| CD38 | CA1 | BLK | GRIA2 |
| CD81 | CA6 | CHRNA7 | MDM4 |
| CDC25A | CA12 | CSK | PCSK7 |
| CDK1 | CA14 | BMX | SMO |
| CDK2 | CA9 | LYN | CCND3 |
| CDK4 | CA5B | TEK | CCND2 |
| CES1 | CA5A | BTK | TKT |
| CES2 | CA3 | FES | TUBB3 |
| CETP | CA4 | TLR7 | RBBP9 |
| CFTR | HCAR2 | PAK4 | MPI |
| CHUK | CA13 | PDPK1 | PITRM1 |
| CNR1 | NQO2 | CAPNS1 | ALPL |
| CNR2 | AKR1C4 | PYGL | HDAC5 |
| CPT1A | FYN | CSNK1A1 | HDAC7 |
| CPT2 | LCK | GHSR | HDAC9 |
| CREBBP | NGFR | CHRM1 | MAP3K8 |
| CTBP2 | TPMT | PIK3R1 | CCR8 |
| CTNNB1 | MAOB | NOD1 | FGFR3 |
| CTRC | PTGS1 | PIP4K2C | PLA2G7 |
| CTSB | F3 | MAP2 | CYP24A1 |
| CTSD | KDM4E | QTRT1 | LSS |
| CXCR1 | KDM4A | FPR1 | INCENP |
| CXCR2 | CYP11B2 | SLC9A1 | TTL |
| CXCR3 | HTR6 | CYP11B1 | IMPDH1 |
| CYP17A1 | TSPO | FLT4 | SLC6A1 |
| CYP19A1 | CTSK | TTK | F11 |
| CYP1A1 | CTSL | THRA | TRPA1 |
| CYP1A2 | GRM2 | F10 | CDC25C |
| CYP26B1 | TRPM8 | KCNH2 | TRPV3 |
| CYP2A6 | GABRB3 | PDE7A | CALCRL |
| CYP2C19 | GABRG2 | TYRO3 | CHRNA5 |
| CYP2C9 | GABRA5 | AXL | DPP8 |
| CYP2D6 | RGS4 | DUT | DPP9 |
| CYP3A4 | FAAH | ADAM10 | CHRNB3 |
| DGAT1 | PPP1CA | TNK2 | CHRNA6 |
| DNMT3A | CMA1 | REN | BAZ2B |
| DPP4 | CTSG | BDKRB1 | BAZ2A |
| DRD2 | TNKS2 | ADRA1D | DPP7 |
| DRD4 | CHRM5 | ADRA1A | RPS6KA1 |
| EGFR | PGR | ADRA1B | CAMK2G |
| ELANE | GABRA3 | CASP1 | WNK2 |
| ENPP2 | GABRA1 | UGCG | MTAP |
| EPAS1 | GRM5 | PDE11A | OGFRL1 |
| EPHX1 | RGS8 | CHRM3 | KCNK2 |
| ERBB2 | ADORA1 | HTR4 | H1F0 |
| ERN1 | PREP | CTSE | LAP3 |
| ESR1 | MGLL | MCHR1 | CBR1 |
| ESR2 | HCRTR2 | PLAT | PLA2G5 |
| EZH2 | HCRTR1 | CCR3 | PLA2G10 |
| F2 | TRPC6 | HSP90AA1 | HSD17B14 |
| FABP1 | TRPC3 | RPS6KB1 | RPS6KA5 |
| FABP2 | S1PR3 | PDE8B | YWHAG |
| FABP4 | RPS6KA2 | HTR1D | CALM1 |
| FAP | GSK3B | ATAD2 | PGF |
| FASN | PDE10A | MAP3K12 | SLC5A4 |
| FBP1 | TBXAS1 | PDE9A | EIF4A1 |
| FDFT1 | PDE4B | SLC5A7 | SLC28A3 |
| FFAR1 | CRHR1 | SRD5A1 | SLC29A1 |
| FFAR4 | CCNE2 | HSD17B3 | AMY1A |
| FGF2 | ADORA2B | SRD5A2 | GRK6 |
| FGFR1 | CTSS | TYMS | SLC22A1 |
| FGR | MEN1 | ACLY | MC1R |
| FLT1 | FNTA | EGLN1 | MC5R |
| FOLR2 | FNTB | ALOX12 | TREH |
| FTO | PDE2A | SLC22A12 | PYGM |
| G6PD | HTR2B | RXRB | FOLH1 |
| GABRA2 | ADRA2C | ITGAL | AMY2A |
| GBA | ADRA2B | ITGB2 | CDA |
| GCGR | DRD1 | CYP26A1 | NAALAD2 |
| GCK | GABRB2 | SLC16A1 | ADA |
| GLUL | HTR2A | RORB | PYGB |
| GPBAR1 | TACR2 | DAGLA | OGA |
| GPR119 | DRD3 | EDNRA | GGH |
| GPR55 | OPRD1 | OXER1 | GLI1 |
| GRK2 | MAPK11 | ALOX15 | ATP1A1 |
| GSTM2 | PGGT1B | TAS2R31 | DHODH |
| GSTP1 | GALR3 | GRIN1 | PPM1B |
| HDAC1 | DYRK1B | EPHB4 | PPP1CC |
| HDAC3 | PABPC1 | HPGD | PPP2R5A |
| HDAC8 | MAPK10 | HRH2 | KCNMA1 |
| HK2 | ADAM17 | SOAT1 | SLC19A1 |
| HMGCR | KCNA5 | ABHD6 | FOLR1 |
| HNF4A | FKBP1A | SLC5A1 | SLC46A1 |
| HPSE | HTR7 | AVPR1A | FPGS |
| IRS1 | MTNR1A | PLK4 | GART |
| HSD11B1 | MTNR1B | CASP2 | ATIC |
| HSPA1A | CLK3 | TAAR1 | GABBR1 |
| ICAM1 | PTPRF | HSP90AB1 | XPO1 |
| IDH1 | PTPN2 | ATP4B | LGALS9 |
| IGF1R | ACP1 | ATP4A | SSTR2 |
| IKBKB | PLA2G1B | PDE4C | SSTR1 |
| IL2 | PDE4D | IRAK4 | SSTR3 |
| IL6 | PTGES | PORCN | FGF1 |
| IL6ST | LTB4R | GRIA1 | SSTR5 |
| INSR | NR3C2 | RPS6KA3 | YARS |
| IRAK1 | FABP3 | PPIA | HSP90 |
| ITGB1 | FABP5 | CSNK1D | LACTB |
| JAK1 | SIGMAR1 | PI4KB | CALM |
| JAK2 | ADORA3 | SAE1 | HAS2 |
| JUN | TOP1 | UBA2 | FASN |
| KDR | PRKCH | CDC42 | KLF7 |
| KIT | PTGER4 | SLC1A3 | KCNA3 |
| LGALS3 | AMPD2 | HTR3A | METAP2 |
| LIPA | GRIK1 | BCAT2 | LTA4H |
| LIPE | GRIK2 | PARP10 | EED |
| LPL | PTGDR2 | CHEK2 | SUZ12 |
| MAOA | PTGIR | CCNC | RBBP7 |
| MAP2K1 | ALOX5AP | AGPAT2 | RBBP4 |
| MAP3K20 | FUT7 | DHFR | PTPRC |
| MAP3K5 | TYR | CHRM4 | BDKRB2 |
| MAP3K7 | COMT | MAP4K4 | NR4A1 |
| MAPK1 | DUSP1 | CBFB | CACNA1C |
| MAPK14 | HSD17B2 | HSD17B1 | SSTR4 |
| MAPK3 | TRPV1 | PNMT | EIF4H |
| MAPK8 | AKR1C3 | RPS27 | FUCA1 |
| MAPK9 | AKR1C2 | GLO1 | HK1 |
| MC4R | AKR1C1 | MMP12 | PNP |
| MCL1 | S1PR1 | EZR | MAP3K9 |
| MDM2 | LPAR6 | NAAA | NUDT1 |
| MERTK | LPAR5 | PGK1 | GYS1 |
| MET | PAM | GAK | TDO2 |
| MGAM | SIRT2 | ILK | CAPN2 |
| MGAT2 | LPAR3 | EIF2AK3 | TACR3 |
| MIF | LPAR2 | GRM1 | KNG1 |
| MMP1 | LPAR1 | PLK3 | NR5A1 |
| MMP10 | LPAR4 | SETD7 | NPEPPS |
| MMP13 | PRSS1 | IDO1 | SLC6A5 |
| MMP14 | PRKCG | TBK1 | PRLHR |
| MMP2 | BACE1 | GABRA6 | CTSH |
| MMP7 | PRKCB | LIMK1 | DDAH1 |
| MMP9 | PSEN2 | HDAC2 | LIPG |
| MPO | PSENEN | PKN1 | SCN2A |
| MTOR | NCSTN | NEK2 | SCN10A |
| NAMPT | APH1A | NEK6 | TNNC1 |
| NCOA2 | PSEN1 | HDAC10 | TNNT2 |
| NFE2L2 | APH1B | NUAK1 | TNNI3 |
| NLRP3 | C5AR1 | ZAP70 | ERBB4 |
| NOD2 | TNKS | EPHA2 | P4HTM |
| NOS2 | LIMK2 | MME | KMT5A |
| NOS3 | CDC7 | EPHX2 | HIPK4 |
| NOX4 | PFKFB3 | MARS | SBK1 |
| NPC1L1 | HRH3 | WEE1 | PHKG2 |
| NQO1 | HRH4 | SCN4A | DAPK3 |
| NR1H2 | F2R | CDK3 | MYLK2 |
| NR1H3 | GRIN2B | OGT | RPS6KA4 |
| NR1H4 | RARG | NR1D1 | PI4KA |
| NR1I2 | RARA | MMP3 | PRKD1 |
| NR1I3 | NPY5R | MMP8 | STK10 |
| P2RX7 | NPY2R | HCK | PHKG1 |
| PARP1 | SLC6A9 | PTK6 | ABL2 |
| PDE4A | QPCT | KCNJ11 | FRK |
| PDGFRB | OXTR | PDCD4 | STK36 |
| PEPD | ASAH1 | DNM1 | TXK |
| PER2 | ABL1 | PRKCZ | EPHA6 |
| PIK3CA | PARP2 | GLI2 | TNIK |
| PIK3CB | PTAFR | PDK1 | SERPINE1 |
| PIK3CD | KIF11 | BMP1 | SHBG |
| PIK3CG | TBXA2R | EP300 | SHH |
| PIM2 | PDE6D | DUSP3 | SI |
| PKM | IMPDH2 | ESRRA | SCN9A |
| PLA2G4A | ITK | ESRRB | BACE2 |
| PLAU | SLC6A15 | EPHB2 | PDE5A |
| PLG | BRD4 | EPHA5 | PDGFRA |
| PPARA | BRD2 | EPHA4 | PLA2G2A |
| PPARD | BRD3 | EPHA8 | KLK2 |
| PPARG | YES1 | EPHA7 | RELA |
| PPP2CA | ATP12A | EPHB3 | LDHB |
| PRF1 | TOP2A | EPHA3 | AVPR2 |
| PRKAA2 | MST1R | EPHB1 | DAPK1 |
| PRKAB1 | ATR | EPHA1 | RXRG |
| PRKACA | JAK3 | MYLK | SCD |
| PRKAG1 | PIM1 | AKT2 | SELE |
| PRKCA | CCKBR | HPGDS | TAOK1 |
| PRKCD | GRK3 | ANPEP | HIPK1 |
| PRKCE | GRK5 | BRD9 | CFD |
| PRKCQ | TNNI3K | TRAP1 | FPR2 |
| PRKDC | DYRK4 | HSP90B1 | PTP4A3 |
| PTGS2 | CDK5R1 | MELK | KLK1 |
| PTK2 | CDK5 | SPHK2 | RXRA |
| PTK2B | DYRK1A | CTSV | HIPK3 |
| PTPN1 | GSK3A | WNT3A | KDM5B |
| PTPN11 | CSNK2A1 | SORD | RORA |
| RAC1 | CSNK2A2 | PDE3A | DYRK2 |
| RAF1 | SLK | PDE3B | CYP1B1 |
| RARB | CLK4 | SMYD2 | RORC |
| RBP4 | CLK1 | SCN5A | DYRK3 |
| RHOA | CLK2 | LDHA | MARK1 |

**Supplementary Table 3. The Targets of NAFLD**

| Name | Name | Name | Name |
| --- | --- | --- | --- |
| PNPLA3 | VEGFA | CYGB | SUCNR1 |
| NFE2L2 | HSD17B13 | ABHD5 | SDF2L1 |
| PPARA | MIR203A | IGF2-AS | CRELD2 |
| ADIPOQ | SPINK1 | MDK | JRKL |
| CYP2E1 | SLC2A1 | PANDAR | NLRC3 |
| LEP | MIR205 | MIR363 | METTL7B |
| TGFB1 | MAPK1 | SRA1 | SLC3A1 |
| FGF21 | TIMP1 | GRK2 | ALK |
| GNMT | MIR15A | CASP4 | BCL6 |
| LDLR | ABCB1 | CYBA | COL17A1 |
| FAS | MIR18A | ITGA5 | MLH1 |
| NR1H4 | CYP2C19 | ZEB2-AS1 | GJB2 |
| PEMT | MIR145 | P2RX7 | DRD2 |
| CAT | MIR143 | ARRB1 | DRD4 |
| ADH1B | CYP2C9 | PER2 | SLC6A3 |
| IL1A | MIR93 | DKK1 | GABRA2 |
| ALDH2 | IL2 | MICA | GCK |
| SREBF1 | MIR125A | ASS1 | IRDN |
| GSTT1 | FGFR1 | MTA1 | LAMC2 |
| SIRT1 | GHRL | CSN1S1 | LAMB3 |
| FOLR2 | MIR34C | NCOA3 | RMRP |
| GSTP1 | NPY | ANGPTL8 | PSMD7 |
| GSTM1 | MIR183 | MIR152 | MYH9 |
| LAMA1 | MIRLET7E | PRDX5 | TACR1 |
| NR5A2 | MIR19A | ABCG8 | SNCA |
| CSF2 | CREB1 | CCR1 | BRAF |
| ACE | NPM1 | DGCR8 | SLC18A2 |
| AHCY | MIR31 | GFER | MECP2 |
| AHR | EGFR-AS1 | ADIPOR1 | NONO |
| PTEN | MIR429 | MIR125B2 | SH2D1A |
| XBP1 | MMP2 | MIR615 | STS |
| PPARD | MIR16-1 | TRPV6 | G6PD |
| SERPINB2 | CDK4 | CCL20 | MTRNR1 |
| PDK4 | ABCC1 | ZNRD1ASP | SLC11A1 |
| LIF | KIT | CCN1 | ABCC8 |
| TM6SF2 | MIR141 | SERPINB3 | TJP1 |
| MMP1 | APP | SNHG6 | SNU13 |
| IL4 | MIR181B1 | LOC110806263 | CTRC |
| IL3 | PRKN | PRPF8 | PSMD4 |
| ALDH1A1 | MIR30D | GATAD2A | MEIS1 |
| TNFRSF1B | TGFBR2 | CXCR1 | TALDO1 |
| PRKCA | ICAM1 | SLC10A1 | NCAPH |
| SCARB1 | CYP19A1 | OSM | CNN2 |
| PRKACA | IGF2R | GHET1 | CFTR |
| CYP17A1 | AHSG | FGFR4 | PSMD9 |
| VLDLR | APOC3 | NOTCH4 | PSMD13 |
| CYP1A2 | PRMT7 | DACT1 | PSMD11 |
| PRF1 | MIR106B | TBP | PSMD12 |
| ABCB4 | MIR181A1 | MIR219A1 | PSMD5 |
| F2 | BAX | ABCG5 | MKKS |
| ALDH1B1 | ABCB7 | MIR211 | HPS1 |
| KLB | HMOX1 | UCP1 | USH1C |
| ABCC2 | ABCB11 | HJV | LAT2 |
| JAK2 | PRKAB1 | S100A9 | NOD2 |
| TRIB3 | NOTCH1 | BRF1 | DNAJC12 |
| ADH1A | RHOA | RRM2 | NCAPG |
| CPT1A | IGF2 | HOXA-AS2 | GBA |
| GSTA1 | CSF3 | TMSB4X | OPN4 |
| ADH4 | EZH2 | APOA4 | HEXA |
| RDX | MIR195 | USF1 | GALT |
| NQO1 | NOS3 | SNHG3 | PSMD14 |
| ALDH4A1 | MDM2 | MIR485 | IL23R |
| B3GAT1 | BIRC5 | ETS1 | TAGLN3 |
| IKBKG | MIR96 | CTAG1B | ASPA |
| EIF2AK1 | CDKN2B | CPS1 | CLN5 |
| PRKCD | PRKAG1 | FDFT1 | MEFV |
| PRKCE | MIR23B | CASP6 | NCAPG2 |
| STC2 | FGF2 | WNT1 | MCCC2 |
| CD14 | CD274 | GLS | PNKD |
| RAG2 | CCN2 | LNCRNA-ATB | NCAPD3 |
| ATP5F1B | TSC1 | MIR216A | CHD8 |
| GPT | LRRC56 | NOX4 | NCAPH2 |
| GABPA | STAT1 | MIR129-1 | PAH |
| TNF | NAMPT | IFNL3 | EID3 |
| SAMM50 | PLIN2 | AQP9 | NSMCE4A |
| HFE | CDKN1B | JPX | AGA |
| MAP3K5 | CYP2D6 | CDH17 | TTC39B |
| NLRP3 | TCF4 | CASC11 | NCAPD2 |
| SLC5A2 | IRF1 | MIR100HG | NSMCE2 |
| MTTP | FABP1 | MIR675 | NSMCE1 |
| TLR4 | SLC25A13 | PRKCQ | PSMD3 |
| XPR1 | MIR127 | MAGEA4 | PSMD1 |
| KRT18 | TSC2 | MIR7-3HG | PSMD8 |
| CD36 | MIR140 | BCS1L | PSMD6 |
| GLP1R | CNR1 | TLR5 | TMED2 |
| INTU | IGF1R | TRIM21 | FXR |
| MAPK8 | DDIT3 | CPB2 | Nrf2 |
| MIR122 | MBOAT7 | UBD | PDE4 |
| IL1B | RB1 | MIR215 | PPAR |
| FGF19 | MIR185 | ZFP36 | BIRC2 |
| MIR21 | TTR | PCAT29 | HSPA5 |
| ADAMTS13 | MIRLET7B | MIR503 | ANXA1 |
| HP | MLXIPL | NPC2 | NPC1L1 |
| HMGB1 | MTR | HDGF | CXCR3 |
| SMARCA4 | HNF1A | PLIN1 | SLC25A47 |
| GPBAR1 | ALDH3A1 | CDKN1C | FFAR1 |
| MIR34A | VCP | CCNE1 | GPS2 |
| HIF1A | MIR373 | SF3B1 | GRB2 |
| HGF | HOTAIR | FAM215A | GRN |
| IGF1 | CCL5 | MAGEA3 | GSTA4 |
| LGALS3BP | MKI67 | PARVB | GSTM2 |
| IL6 | FOXP3 | CTBP2 | TNC |
| PPARG | GNAS | PALLD | IDH2 |
| CXCL10 | MIR499A | STUB1 | IFNB1 |
| LGALS3 | MIR27B | AIFM1 | IL18 |
| IL17A | MIR25 | LEPROT | INS |
| FOXO1 | BMP6 | MAP3K7 | INSR |
| RARRES2 | RARB | CNR2 | IRAK1 |
| DPP4 | NFKB1 | GPD1 | IRF7 |
| SOD2 | MIR224 | HPSE | ITGAX |
| CFLAR | MIR30A | RSPO3 | ITPR2 |
| MIR223 | CREBBP | MST1 | ENHO |
| RBP4 | DGAT1 | SFRP1 | IL13RA2 |
| AR | TFAP2A | MAPK9 | IL13 |
| ACAT1 | SHC1 | GOT1 | IFNG |
| LBP | FOS | CTTN | ARSH |
| JUN | NEAT1 | ACTA2-AS1 | APOE |
| GTF2H1 | AGT | NDUFA13 | IL1R1 |
| MIR33A | SERPINA1 | IFNL4 | IL1RN |
| MAT1A | PLAU | MAGEA1 | IL6ST |
| CEACAM1 | MIRLET7A3 | RXRG | CXCR2 |
| MT1B | H19 | CBR3-AS1 | IL10 |
| CMKLR1 | MIR372 | SALL4 | MALAT1 |
| MIR144 | PSMD2 | TPX2 | UGT1A1 |
| IFNA13 | DIABLO | ADD1 | SLC22A6 |
| APOB | MGMT | ZEB1-AS1 | SLCO1B1 |
| MIR192 | KRT8 | TCF7 | SLCO1B3 |
| ST3GAL4 | DANCR | IL36A | SLCO2B1 |
| SQSTM1 | XIAP | BHMT | ALB |
| SLC10A2 | LMNA | GAS5-AS1 | KRAS |
| IFNA1 | GCKR | CPS1-IT1 | MIR10B |
| PNPLA2 | MIR148A | PODXL | GGT1 |
| CXCL8 | CASP9 | ORM1 | NAFLD1 |
| CCL4 | BIRC3 | MECOM | LEPR |
| MIR451A | CDK1 | MAGEC2 | MSH2 |
| SYK | SCD | KLRK1 | ERBB2 |
| TAZ | SHBG | MAD2L1 | SLC6A4 |
| PIN1 | MEG3 | MIR340 | IRS1 |
| NR0B2 | FABP2 | PCBP2-OT1 | NAFLD2 |
| TP53 | MIRLET7G | MIR301A | AKT1 |
| CCR2 | HBA1 | RIOX2 | MIR155 |
| UCP2 | MIR23A | FST | MET |
| CASP8 | PSMD10 | MGAT5 | CDKN2A |
| VTN | PVT1 | NPTN-IT1 | HRAS |
| AEBP1 | MIR137 | MIR374A | CTNNB1 |
| NUP62 | BCL2L1 | DRAIC | MIR17 |
| DECR1 | ITGB1 | SNTA1 | MIR126 |
| SERPINF1 | MIR196A2 | NANOG | MYC |
| AGER | HULC | MIR26B | BRCA1 |
| FABP4 | MIR191 | WT1-AS | NRAS |
| DCTN4 | CYCS | APOA2 | BCL2 |
| PKM | MIR128-2 | EEF1A1 | MIR200B |
| KHDRBS1 | PPIG | DYSF | STAT3 |
| CRP | PTK2 | ARHGEF40 | FASLG |
| MPO | CAV1 | ASPH | LPL |
| GPX1 | CXCL12 | LIN28B | SERPINE1 |
| MC4R | LEPQTL1 | HOXA13 | MIR29C |
| MMP9 | NOS2 | SUFU | SOD1 |
| GPR119 | ERBB3 | CXCL9 | CCND1 |
| TLR9 | JAK1 | NCAN | SRC |
| GGTLC1 | ENPP1 | ATF6 | EGF |
| CD40LG | DGAT2 | RORC | PTGS2 |
| PON1 | PCNA | SOST | MAOA |
| RETN | SMAD3 | PRAL | MIR146A |
| CD44 | HSPA4 | MIR490 | APC |
| LGALS14 | MIR9-1 | CERNA2 | MIR222 |
| MYOM2 | PARP1 | CRABP2 | CYP1A1 |
| IL20RA | APOA5 | MIRLET7A2 | APOA1 |
| PTPN11 | PRKAA2 | PINX1 | MIR27A |
| MIR182 | CPT2 | IFNAR2 | TERT |
| SETMAR | CDK2 | SUGP1 | CTLA4 |
| CD1D | CP | SF3B2 | MIR200A |
| PLG | DNMT1 | ATP8A1 | MIR200C |
| FBXW7 | IL6R | USF2 | CDH1 |
| PRX | BGLAP | PDGFA | MAP2K1 |
| KEAP1 | KDR | MIR124-2 | CYP3A4 |
| PIK3CG | MIR26A1 | ABO | SLC2A4 |
| FBXW5 | VCAM1 | RB1CC1 | TF |
| CD40 | DNMT3A | EDA | MIRLET7C |
| MAPK3 | POLG | STEAP3 | MIR20A |
| PSPH | FTO | PHF20 | MIRLET7D |
| AKR1B10 | CALCA | SLC4A2 | MIR22 |
| CD38 | RAF1 | RSF1 | MIR130A |
| MTDH | CDKN2B-AS1 | DBH-AS1 | LIPC |
| GDF15 | EPCAM | ARG1 | CYP2A6 |
| LITAF | BANCR | HTATIP2 | MIR107 |
| ZC4H2 | PDGFRB | GPLD1 | CASP3 |
| H4C15 | VIM | GPNMB | CDKN3 |
| SLC12A9 | TREM2 | CACNA2D1 | HNF1A-AS1 |
| AZGP1 | GAPDH | FAH | MVP |
| PTPN1 | CYP2C8 | YARS1 | DGCR5 |
| CCL4L2 | ZEB1 | TP53BP2 | CD80 |
| JCAD | NR1H3 | SLC8A1 | CDC25A |
| MOCOS | SP1 | RPTOR | LINC02055 |
| AVP | LINC-ROR | MIR103A2 | TIMP3 |
| SEMA6A | TGFA | MAP3K20 | AOX1 |
| MIR146B | SERPINA3 | DYNLRB2 | ACADM |
| PTPA | GSR | RHOC | LINC00926 |
| KLF4 | MIR181A2 | C1orf94 | LINC00974 |
| H4C3 | FHIT | CYP2J2 | PINK1 |
| SUV39H2 | PDGFB | CBX4 | HSD11B1 |
| CCK | GAS5 | C20orf204 | JAG1 |
| CXCR4 | MIR30E | SMURF1 | LINC00554 |
| PLA2G6 | CCNA2 | SERPINA12 | LINC00601 |
| STK24 | ALPP | FTH1 | MIR101-1 |
| TNFRSF4 | MIR151A | NNT-AS1 | MIR324 |
| TRAF6 | SET | STING1 | IL11 |
| TNFRSF1A | MCL1 | RIPK1 | ABCC3 |
| TNFAIP3 | ADRB2 | MAPKAPK5-AS1 | IL12RB1 |
| TLR3 | LPA | FER1L4 | BID |
| TLR2 | MIR181C | TNFRSF12A | MMP14 |
| THRB | CCNB1 | MIR9-2 | PHGDH |
| TGM2 | TFRC | COPS5 | PKLR |
| HAVCR2 | TGFBR1 | ABCG1 | LRP2 |
| RUNX1 | ABCG2 | PYCARD | DROSHA |
| GSDMD | RPS27A | ESM1 | SLC27A4 |
| EHMT1 | MIR24-2 | PPP2CA | APOC2 |
| CALCR | ANXA5 | PIM2 | ADRB1 |
| H4C8 | CHUK | DYNLRB1 | HDAC8 |
| H4C11 | MIR214 | FGL1 | SLC40A1 |
| H4C2 | FN1 | TREM1 | ACER3 |
| H4C12 | C3 | PCNA-AS1 | FTL |
| H4C6 | ADIPOR2 | GRAMD1A | AURKA |
| H4C4 | MIR204 | INSIG2 | SERPINB5 |
| H4C1 | LINC00261 | ST6GAL1 | TJP2 |
| H4C9 | TNFRSF11B | SLC9A9 | WNT5A |
| ARID1A | H2AC18 | MT-CYB | WRAP53 |
| BAS | NORAD | PEG10 | CD81 |
| H4C5 | NPPB | NCOA1 | PTX3 |
| H4C13 | AXIN1 | ARHGEF39 | AURKB |
| MADCAM1 | PTK2B | MIR1-2 | RDH5 |
| H4C14 | NUS1 | CDH4 | ENG |
| STAM | KRT19 | ACACB | CPQ |
| PLPP3 | PROM1 | MIR491 | SNHG15 |
| RUNX3 | UCA1 | DLC1 | NRP1 |
| SRSF3 | EDN1 | LATS2 | BECN1 |
| SFRP5 | MIR138-1 | PRDM2 | SNHG20 |
| HDAC3 | PDCD1 | MIR136 | CLOCK |
| ELOVL6 | XIST | HSPA6 | DNASE1 |
| SELENOP | NFKBIA | PTENP1 | POSTN |
| MGAM | EPO | FGF3 | MIR133A1 |
| CXCL5 | GNRH1 | FAM83D | FOXO3 |
| CCL2 | RAC1 | GREM1 | MSBP1 |
| RORA | CES1 | FBP1 | SLC22A5 |
| BCL2A1 | TUG1 | C14orf132 | CD82 |
| IL33 | IFNA2 | CCRL2 | NR1I2 |
| SART1 | NR1H2 | ZNF638 | FTX |
| MOK | HAGLR | IRX3 | MIR206 |
| RAB5A | GPI | PANX1 | PCK2 |
| BCAT1 | MIR378A | IATPR | SAA1 |
| XPO4 | MSR1 | PSG1 | SOX4 |
| SHH | ST3GAL3 | MMP11 | GOLM1 |
| SHMT1 | CDH2 | MAU2 | CD163 |
| TFF3 | EPHX1 | INHBA | TFEB |
| TMBIM6 | EGR1 | RBM39 | SNHG12 |
| TCF7L2 | MIR95 | HSALNG0013825 | TP53BP1 |
| MIR33B | NME1 | CCR9 | CD68 |
| IRS2 | HEIH | BAIAP2-DT | MIR130B |
| STK11 | AREG | E2F2 | TRIM8 |
| TNFSF10 | PkOU5F1 | MIR500A | STMN1 |
| SREBF2 | EIF2AK2 | NRG4 | CES2 |
| TNFRSF6B | MTUS1 | CAB39 | ACOX1 |
| TNFRSF10B | CCR6 | CECR7 | MERTK |
| SPP1 | PTCH1 | TFDP1 | IFNAR1 |
| SMPD1 | DEPDC5 | CCEPR | LINC01684 |
| SMARCA1 | ALOX5 | SCO1 | LEF1 |
| SLCO1A2 | MIR149 | UBB | SNAI2 |
| SI | ACTC1 | EIF2D | MIR22HG |
| HAMP | UGT1A7 | ZNF674-AS1 | ATP8B1 |
| DDC | EIF4EBP1 | LPIN1 | REG3A |
| ELN | MIR424 | MIR26A2 | PEPD |
| SLC51A | CBS | CIDEC | GPC3 |
| FBL | GLUD1 | LAPTM4B | IGFBP2 |
| ESR1 | GLUL | LECT2 | MIR15B |
| ESR2 | SULT2A1 | COL13A1 | KLF6 |
| ACSL4 | CSF1 | RAB4B-EGLN2 | CASC15 |
| FAP | MUTYH | ITGA8 | BSG |
| FASN | MIF | TIFA | GPX3 |
| ELANE | UCHL1 | TSLP | ATP7B |
| MLKL | FLT1 | ERN1 | AOC3 |
| GADD45A | EPAS1 | LDC1P | FOXM1 |
| AFP | SPARC | GNAI2 | TP53COR1 |
| DNMT3B | MIR154 | SH2B1 | NKILA |
| ATN1 | HBB | TWIST2 | MIR24-1 |
| AGTR1 | PDGFRL | MIR876 | STARD13 |
| E2F1 | MIR150 | SLC30A10 | MIR30C1 |
| ABCA1 | HOTTIP | GPR55 | FZD7 |
| EGFR | ENO1 | CHP2 | TP73-AS1 |
| FAT1 | IKBKB | ANGPTL3 | CIDEB |
| FGR | XDH | UFM1 | CNOT9 |
| AKR1B1 | TWIST1 | ACADVL | KCTD13 |
| TRPC4AP | H6PD | SULF1 | ECI2 |
| GCG | LIVAR | FRZB | HINT2 |
| GCGR | MIR199A1 | MT-ND6 | PRAP1 |
| SLC17A5 | TXN | TRIM33 | DNAJA3 |
| GDF2 | MIR483 | LARP1 | RNF114 |
| RNU1-1 | SELE | LOXL1 | EHBP1L1 |
| GH1 | MIR181B2 | ATF4 | TMEM51-AS1 |
| GHR | HDAC1 | CYP26B1 | MRTFA |
| IBTK | DPYD | DMGDH | OVCH1-AS1 |
| WWTR1 | IDH1 | UBE2D2 | SEMA6A-AS1 |
| DICER1 | ANGPT1 | USP7 | CA3-AS1 |
| SIRT3 | TIMP2 | WDR1 | PRC1-AS1 |
| MLYCD | CD34 | UFC1 | THY1-AS1 |
| MTOR | PHB1 | CDR1 | NUPR1 |
| FNDC5 | ACTB | SAPCD2 | MAT2A |
| PCSK9 | MIR142 | JAZF1 | VPS37A |
| ZGLP1 | LINC00210 | SERHL | SPRTN |
| HSPA12A | LINC00173 | SH3BP5 | DUSP26 |
| GIP | SOX2-OT | YWHAZ | SEL1L3 |
| MIR873 | PRKAA1 | PDGFC | SESN2 |
| TNFSF13B | CCAT1 | NDRG2 | IL19 |
| CETP | ANGPT2 | MACROD2 | USP18 |
| NMU | ZEB2 | ELK1 | TFDP3 |
| PPARGC1A | VEGFC | SMYD3 | NAA40 |
| RIPK3 | SKP2 | PZP | PRPSAP1 |
| ADAMTS5 | G6PC1 | MIRLET7F2 | FAM83A-AS1 |
| CHIT1 | ACTA2 | PFKFB1 | TOMM20 |
| RASSF1 | CCAT2 | OSMR | COX6B1 |
| TXNIP | MIR187 | POLR2D | MCU |
| PRDX4 | PRECSIT | lnc-TM6SF2-1 | VNN1 |
| LRPPRC | TNFRSF10A | piR-37045 | ZDHHC2 |
| LPCAT3 | FALEC | HSALNG0135823 | PLIN5 |
| CDKN1A | HK2 | TUBA8 | PHF2 |
| LOC102724197 | KCNQ1OT1 | LOC121627950 | FGL2 |
| AKR1A1 | COL3A1 | CHST2 | LPIN2 |
| TLR6 | PLAUR | NR2F1-AS1 | HEPN1 |
| YAP1 | TFR2 | URGCP | CARD8-AS1 |
| NCOA2 | KRT7 | LCN1 | SLC23A1 |
| LNCARSR | CASP7 | EIF2S1 | NOTUM |
| CLCN2 | HSPB1 | GLYAT | TCP10L |
| CLU | SMAD2 | HTR2A-AS1 | PDIA3P1 |
| MAPK14 | MIR139 | ZNF350-AS1 | SIRT1-AS |
| SESN3 | MIR125B1 | MIR1-1 | GPC3-AS1 |
| TICAM1 | CAMTA1 | PSD3 | CREB3L3 |
| ADRA2A | MIR196A1 | SLC7A6 | TMEM176A |
| CTSB | PRDX1 | CCL19 | C9orf78 |
| CTSD | NR1I3 | TMBIM1 | DIP2C-AS1 |
| ADRB3 | SOCS1 | SCAP | DEFA1 |
| CYP2B6 | SNHG1 | CXCL16 | ZNF267 |
| TRIM69 | SOS1 | TRPV5 | DNAJC15 |
| ATF2 | HSPA1A | AOC4P | MYCT1 |
| H4-16 | PRKDC | BMP8B | CFAP52 |
| CCR5 | MIR124-1 | CLDN10 | PLPP5 |
| ADH1C | SLPI | GDF11 | FABP5P3 |
| COL1A1 | CEBPB | TRA2B | PERCC1 |
| BHLHE23 | CXCR5 | RBP5 | ULK4P2 |
| PPARGC1B | PLA2G4A | VLDLR-AS1 | CCND3P1 |
| ADORA2A | LINC00665 | LTBP3 | NARF-AS1 |
| CPA1 | LINC00941 | TGS1 | CXCL1P1 |
| AWAT1 | MIR148B | STEAP4 | FFAR4 |
| NOX1 | LOX | ST8SIA1 | UBXN1 |
| RTL1 | MIR186 | BBC3 | INSIG1 |
| MIR29A | PIGR | ATP5F1E | CIDEA |
| MIR296 | MIR212 | DLGAP5 | NFE2L1 |
| SMAD4 | CEBPA | IL27 | ECH1 |
| SMAD7 | THBS1 | ULK1 | FADS3 |
| SMCP | RXRA | MIR518D | DOK1 |
| MGAT2 | LINC00383 | RAB11FIP4 | RNU6-53P |
| MAP3K11 | MIR99A | MT1G | HERC4 |
| MMP10 | CYP7A1 | MT1DP | SORBS3 |
| MIR221 | ALMS1 | AUP1 | TIRAP |
| CYBB | MIRLET7A1 | URB2 | SOCS7 |
| CCL4L1 | MMP7 | ARC | RREB1 |
| LCN2 | CYTOR | HEPACAM | TLL1 |
| LIPA | LINC01138 | PSME2 | TNIP3 |
| LOXL2 | ZFAS1 | UBQLN4 | FBXO30 |
| LRPAP1 | CD28 | URI1 | AATF |
| ARNT | LIPE | ESRP2 | SLC46A3 |
| LUM | LINC00589 | ZP4 | SLC17A9 |
| MIR132 | LINC02027 | VSIG4 | DDX60L |
| MTHFR | CASC2 | BATF2 | CRACR2A |
| MUC1 | IGF2BP2 | GIHCG | MXD3 |
| MYD88 | TP73 | PTPRH | MSBP2 |
| OLR1 | LINC01018 | AZIN1 | TEX36 |
| IL22 | LINC01036 | TRIM47 | TLCD4-RWDD3 |
| PDE4A | LINC01037 | SNORD138 | RWDD3-DT |
| SIRT6 | COG2 | HS3ST1 | LOC643339 |
| PF4 | MIR199B | lnc-ARHGEF40-2 | SLC23A2 |
| PHB | SOCS3 | HSALNG0099859 | SLC44A1 |
| PIK3CA | CYLD | HSALNG0015893 | ONECUT2 |
| PIK3CB | MIR193A | HSALNG0015894 | SLU7 |
| MIR375 | CCL3 | lnc-SMIM12-1 | SLC27A5 |
| CISD2 | AFAP1-AS1 | piR-45035-016 | FAM3A |
| ATM | LINC01419 | HSALNG0124715 | FAM3B |
| ACO1 | ANXA2 | TTC36 | SLC27A2 |
| NM | GCLC | SLC25A27 | RGS5 |
| NNMT | SNAI1 | KRTAP5-AS1 | JMJD1C |
| NPPC | MIR331 | ALDOB | HMGCR |
| SLC11A2 | MMP13 | YY1AP1 | FOXA3 |
| NRF1 | TYMP | CXCR6 | HNF4A |
| NTS | PCAT1 | EGILA | MIR338 |
| PIK3CD | TUSC7 | YIPF1 | HOXA11-AS |
| OR10A4 | SLC39A8 | SAMD9L | CRNDE |
| HCFC1 | SPRY4-IT1 | LNCBRM | GLT1D1 |
| ANXA6 | LCAT | RCHY1 | FARP1 |
| ACACA | ENPP2 | ARRDC3 | LOC105369388 |
